# Recovery-Limiting Signal-Conditioned Action Decoding for Emergency Wireless Access–Backhaul Capacity Allocation

**DOI:** 10.3390/s26165317

**Published:** 2026-08-21

**Authors:** Jingxiang Ma, Minglei Han, Hongbin Ma, Ying Jin, Youzhi Zhang

**Affiliations:** 1 School of Automation, Beijing Institute of Technology, Beijing 100081, China; 3220215147@bit.edu.cn (J.M.); mingleihan@bit.edu.cn (M.H.); jinyinghappy@bit.edu.cn (Y.J.); 2 Marine Science and Technology Domain, Beijing Institute of Technology, Zhuhai 519088, China; 3 Centre for Artificial Intelligence and Robotics, Hong Kong Institute of Science & Innovation, Chinese Academy of Sciences, Hong Kong, China; youzhi.zhang@cair-cas.org.hk

**Keywords:** emergency wireless, access–backhaul capacity allocation, recovery-limiting signals, structured action decoding, reinforcement learning

## Abstract

Post-disaster emergency wireless access–backhaul recovery requires limited capacity to be allocated across affected regions under nonnegativity and fixed-total-capacity constraints. We propose Recovery-Limiting Signal-Conditioned Action Decoding (RLS-CAD). Critical-region access outage, backhaul shortfall, traffic backlog, and end-to-end recovery deficit form regional score bases, while a 15-dimensional policy controls signal fusion, score correction, and finite-support simplex projection. Experiments use five training seeds and 100 shared test scenarios and retain both training and scenario variability. RLS-CAD outperforms Graph-PPO and both information-matched controls on FinalCap, CTI, and TaskUtility. The evaluation concerns normalized planning-level simulation; physical-layer scheduling is outside its scope.

## 1. Introduction

With observable access-outage, relay-shortfall, queue-backlog, and recovery-gap indicators, emergency wireless access–backhaul capacity allocation requires constrained sequential decision-making and structured action generation. Radio resource management surveys similarly emphasize adaptive wireless resource control under heterogeneous traffic and network constraints [1,2]. A planner must allocate limited resources across many candidate regions, maintain feasibility at each decision period, and account for the delayed effect of current allocations on subsequent demand, congestion, and regional connectivity. Reinforcement learning (RL) can address such sequential decision problems, but standard dense action heads and static priority rules often leave a gap between learned regional scores and executable allocations when the effective action support is small and state dependent [3,4,5]. Emergency wireless access–backhaul recovery provides a planning-layer setting in which a fixed equivalent radio-capacity allocation must be distributed across affected regions, while access interruption, backhaul shortfall, queue backlog, and end-to-end recovery gap determine whether a capacity placement can improve recovery.

In a disrupted access–backhaul recovery chain, the value of a regional capacity allocation depends on the location of the current recovery constraint. Allocating capacity to a priority access area can restore immediate access, allocating it to a backhaul relay can restore connectivity for dependent remote-demand areas, and allocating it to a queue-heavy general area can reduce persistent backlog. Air-to-ground communication planning further shows that temporary wireless support must jointly consider coverage, scheduling, and communication-resource constraints [6]. The same local demand can therefore have different system value depending on whether end-to-end support is limited by upstream relay or backhaul support, nearby access, or traffic backlog. In this setting, the current access–backhaul indicators serve as action-decoder context. The decoder must convert access-outage, relay/backhaul-shortfall, queue-backlog, and recovery-gap maps into policy-controlled regional action scores. A feasible regional capacity-allocation action must therefore respect recovery-chain connectivity, local demand intensity, and fixed total-capacity conservation within the same decision step.

Existing resource-allocation methods address parts of this requirement but do not specify how observable recovery indicators should control a feasible fixed-capacity allocation decoder. Access–backhaul and integrated access and backhaul studies show that access demand and upstream support share constrained wireless resources [7,8,9]. Graph neural networks (GNNs) can encode regional dependency structure [10,11,12]. Simplex and sparse projection can enforce fixed-total-capacity feasibility after a policy emits unconstrained regional scores [13,14]. Latent decoders and graph policies are appropriate when relevant structure is hidden, while static recovery-signal coefficients only express fixed allocation preferences. The remaining decoder-design question is how to expose computable access, upstream support, backlog, and recovery-gap maps as score bases while leaving gate selection, score shaping, residual/cost correction, and finite-support projection under actor control.

Recovery-Limiting Signal-Conditioned Action Decoding (RLS-CAD) addresses this gap by converting recovery-limited access–backhaul state into a fixed-total-capacity regional capacity-allocation action. The four signals are the computable current-step combination of access outage, relay/backhaul shortfall, queue backlog, and end-to-end recovery gap on the regional map. RLS-CAD treats these maps as actor-conditioned capacity-placement score bases; the executable allocation is produced only after learned gate selection, score concentration, residual/cost correction, and projection control. The projection layer converts the actor-conditioned net regional score map into a nonnegative, total-capacity-conserving allocation with sparse positive support. The resulting pipeline separates signal construction, learned decoder control, finite-support projection, and the next-state reward/loss signal.

The contributions are threefold:

- A regional wargame-map formulation for emergency wireless access–backhaul capacity allocation with observable recovery signals, drawing on analytical wargaming and modeling-and-simulation practices that organize spatial roles, constraints, options, and consequences for scenario-based decision analysis [15,16]. The formulation maps affected regions, recovery pressure, capacity constraints, and access–backhaul support relations into a repeatable planning-layer representation and formulates the task as a Markov decision process (MDP) with fixed-total-capacity regional capacity-allocation actions.
- A recovery-signal-conditioned RLS-CAD approach in which normalized access-outage, relay/backhaul-shortfall, queue-backlog, and recovery-gap maps provide regional score bases, the actor outputs low-dimensional decoder controls, and the final finite-support allocation is produced through actor-conditioned basis fusion, residual/cost correction, and simplex projection.
- Overall comparisons, controlled ablations, parameter-sensitivity tests, and cross-size evaluations validate the proposed method. RLS-CAD significantly outperforms Graph-PPO and consistently produces finite-support allocations that satisfy the fixed-total-capacity constraint.

## 2. Related Work

Resource allocation under observable recovery constraints and constrained sequential allocation provide the closest task context. In wireless and networking surveys, RL is commonly used to adapt bandwidth, power, channel, or scheduling decisions to traffic and channel states [1,2]. Emergency and public-safety studies operationalize recovery indicators by modeling surviving access resources, temporary relays, and disrupted-area coverage [17,18]. Critical-node and unmanned-aerial-vehicle public-safety work further connects resource placement with connectivity priority and coverage under damaged infrastructure [19,20]. These studies motivate recovery-aware placement, but their decision variables often remain deployment, coverage, power, or link-level scheduling variables. The planning-layer task considered here treats access-outage, relay-shortfall, backlog, and recovery-gap maps as normalized access–backhaul chain pressure and asks how an RL action decoder should transform those maps into a fixed regional capacity allocation.

Structured and gated action representations are needed when feasible allocations must satisfy nonnegativity, fixed-sum, and positive-support sparsity constraints. Standard actor–critic backbones such as proximal policy optimization (PPO), soft actor–critic (SAC), and twin delayed deep deterministic policy gradient (TD3) can be paired with continuous action heads [3,21,22]. Parameterized-action and hybrid-action methods use compact continuous controls to modulate larger structured action spaces [23,24]. Recent model-based parameterized-action work further shows how learned controls can condition discrete–continuous action choices [25]. Dirichlet policies represent simplex-valued allocations stochastically [4]. Euclidean simplex projection and sparsemax map unconstrained scores to fixed-sum vectors and can set low-scoring entries exactly to zero [13,14]. Autoregressive constrained allocation methods embed feasibility into the action-generation order [5]. These methods address structured actions, sparse positive-support sets, or constrained projection, while explicit control of regional capacity-placement score maps through critical access support, upstream support, backlog, and end-to-end recovery limits remains a distinct decoder-design problem.

Access–backhaul and integrated access and backhaul (IAB) resource allocation explain why upstream support can limit downstream connectivity at the planning level. The 3GPP IAB standardization work and early IAB studies identify the coupling between access demand and wireless backhaul resources [7,26,27]. Recent planning-oriented studies treat bandwidth sharing and access–backhaul resource coupling as allocation variables [8,9]. Mobility-aware IAB allocation and RL-based scheduling further show how upstream support constraints affect downstream allocation decisions [28,29]. These works usually optimize link, bandwidth, or scheduling variables and may include physical-layer or medium-access details. The present formulation abstracts upstream-support constraints into planning-layer recovery indicators and represents execution details through equivalent regional capacity allocations.

Graph resource allocation and latent-structure learning are closely related to explicit recovery-signal decoding. GNNs encode node, edge, and topology dependencies for wireless resource allocation and heterogeneous wireless networks [30,31]. Vertex–edge and heterogeneous-attention variants refine how regional or link features enter graph policies [10,32]. Graph RL for routing and combinatorial optimization shows how learned policies can exploit topology-dependent value structure [33,34]; recent wireless mesh studies similarly combine GNNs and DRL for routing and resource allocation [12]. A recent wireless GNN survey emphasizes the representation role of graph encoders, whereas downstream action-decoding constraints still require allocation heads and feasibility mappings [11]. For the present task, graph encoding provides regional dependency features, latent decoding is appropriate when regional connectivity roles must be inferred from data, and projection mappings enforce simplex feasibility. Explicit signal-conditioned decoding adds a different requirement: the policy must control how computable access, upstream support, backlog, and recovery-gap maps shape the regional score map before sparse fixed-total-capacity projection.

Recent studies also indicate three relevant extensions. Channel-model-driven optimization provides a basis for aerial–terrestrial 6G planning [35]. Federated and dynamically updated digital twins can support predictive regional states [36,37,38]. Physical-layer-security games further motivate explicit treatment of malicious interference and untrusted relays [39,40,41]. These extensions are outside the present normalized planning benchmark and are identified as future work.

## 3. Task Modeling and Sequential Decision Formulation

### 3.1. Task Definition

Emergency wireless access–backhaul restoration must allocate scarce planning-layer capacity across affected areas when the remaining wireless infrastructure cannot satisfy all requests simultaneously. The map distinguishes supply-side regions from demand-side regions. Supply-side regions include a network entry that still reaches the core network, temporary access points that cover nearby users, and backhaul relays that carry traffic toward dependent areas. Demand-side regions include priority access areas that require immediate connection continuity, remote-demand areas that depend on upstream relays, and general affected areas where local traffic queues accumulate. Capacity assigned near a temporary access point improves nearby access, capacity assigned to a backhaul relay can restore downstream communication for several regions, and capacity assigned to a queue-heavy general area reduces accumulated traffic only when the access–backhaul support chain is sufficiently connected.

Several recovery constraints can therefore compete for the same fixed capacity allocation. A remote-demand area can exhibit high unmet demand, while the most effective allocation remains an upstream backhaul relay, and a priority access area can require direct allocation even when global queue pressure is concentrated elsewhere. The planner must identify whether access, upstream support, end-to-end continuity, or accumulated backlog is limiting recovery, because improving one local indicator may leave the main constraint unresolved. At the planning layer, each decision is one regional allocation plan. Region roles determine whether capacity mainly supports nearby access, relay or backhaul operation, or remote demand. The continuous quotas can later be mapped to scheduling decisions according to traffic associations and support dependencies, while the present problem retains only the region-level allocation.

### 3.2. Regional Wargame-Map Representation

**Figure 1:**
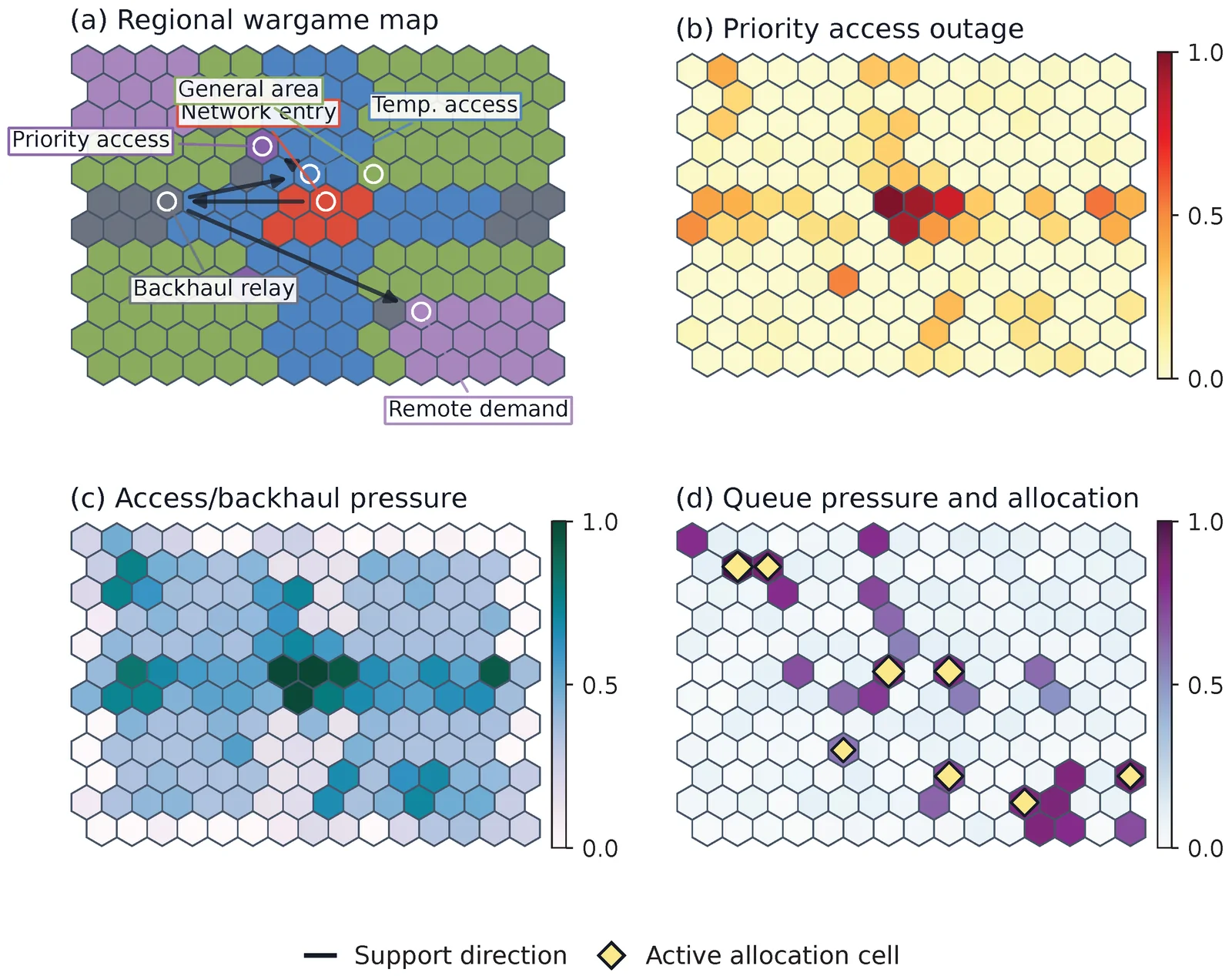
Regional wargame-map formulation for planning-level emergency wireless access–backhaul capacity allocation. Regional cells denote areas that can receive capacity allocation. (**a**) Regional role map; cell colors distinguish the six regional roles identified by the callouts: network entry, temporary access point, backhaul relay, priority access area, remote-demand area, and general affected area. Arrows indicate access–backhaul support direction, and white-ring markers identify the labeled examples. (**b**) Priority-area access interruption intensity. (**c**) Relay/backhaul shortfall and end-to-end recovery pressure. (**d**) Traffic queue pressure and active allocation cells under the same fixed total capacity. Colorbars report normalized within-panel planning-layer intensity.

The affected area is represented using a regional wargame-map abstraction so that access–backhaul recovery constraints can be analyzed without reproducing every link-level scheduling detail. This modeling choice is motivated by analytical wargaming and modeling-and-simulation practices, where spatial roles, scenario constraints, decision options, and simulated consequences are organized on a structured map for decision analysis [15,16]. In the present task, each hexagonal cell in Figure 1a is a regional unit that can be observed and assigned a share of the fixed capacity allocation. The colored cells and direct text labels place the supply-side and demand-side roles from the task definition onto the same planning map. Network-entry and temporary-access labels mark the surviving or deployable access resources, relay labels mark upstream support regions, and priority-access, remote-demand, and general-affected labels mark the main demand regions. White-ring markers identify the role examples named on the map, and arrows show how access–backhaul support flows from entries and temporary access points toward dependent regions. These visual elements define the planning-layer access–backhaul support chain on which recovery-pressure overlays are interpreted.

The same map then serves as the base layer for recovery-limiting signal overlays. The warm pressure field in Figure 1b represents priority-area access interruption, where higher intensity means that nearby users are more severely disconnected and direct allocation can have higher immediate value. The support-pressure field in Figure 1c represents upstream relay or backhaul shortfall together with end-to-end recovery pressure. Darker cells indicate regions whose recovery is limited by relay or backhaul dependency, so an allocation assigned to an upstream relay can affect downstream regions as well as the relay cell itself. The queue-pressure field in Figure 1d represents accumulated traffic backlog, and yellow diamond markers identify cells that receive effective allocation in a representative fixed-total-capacity action. The colorbar in each pressure panel reports normalized within-panel planning-layer intensity for regional comparison. Regional cells, role labels, support-direction arrows, heatmaps, colorbars, and active-allocation markers jointly explain how access-outage, relay/backhaul-shortfall, queue-backlog, and end-to-end-recovery indicators become the explicit recovery-limiting signals used by the decoder.

The hexagons in Figure 1 are visualization units rather than calibrated radio footprints. The implemented benchmark uses a four-neighbor lattice for $E^{\mathrm{sp}}$; route and support edges are constructed from functional roles and nearest eligible providers. Consequently, cell side length is not asserted to equal a coverage radius. In deployment, a cell should be smaller than the spatial scale over which association and link quality change materially, and $E^{\sup}$ should be rebuilt from measured or predicted association, reachability, and backhaul-support records. If a cell is comparable to or larger than a coverage radius, one binary support edge can hide intra-cell coverage holes; if it is much smaller, binary edges can exaggerate boundary changes and increase the planning dimension. This is the planning-to-physical “quantization gap.” The present transition model averages over SINR, interference, fading, and scheduler contention, so its support relations are valid only as scenario-defined functional dependencies, not as a link-level channel model.

### 3.3. MDP Formulation

Capacity allocation changes the subsequent recovery state. We therefore represent the process as a finite-horizon controlled Markov process on a typed regional graph,

(1) $$G=(V,E^{\mathrm{sp}},E^{\mathrm{route}},E^{\sup}),\;N=|V|,$$

where *V* denotes functional regions. The three edge types describe spatial adjacency, service routing, and upstream support. Regional observations combine static role and location attributes with the current communication state, traffic pressure, and previous allocation. The global observation summarizes decision time, system capability, and current recovery loss. The policy observation is

(2) $$s_{t}={\left\{\left(x_{i},d_{t,i},a_{t-1,i}\right)\right\}}_{i=1}^{N}\cup \left\{\psi_{t}\right\},$$

where $\psi_{t}$ is the global state vector. This observation is an approximately sufficient descriptor under the fixed disturbance profile and deterministic default transition used here. Hidden long-term correlations in arrivals or link failures would instead produce a partially observable process, which could be addressed through state-history encoding or belief-state modeling.

At each step, the action is a regional capacity-allocation vector

(3) $$a_{t}=\left(a_{t,1},\ldots ,a_{t,N}\right),\;a_{t,i}\geq 0,\;\sum_{i=1}^{N}a_{t,i}=B,$$

where *B* is the total planning-layer equivalent radio-capacity allocation available in the current period. For a fixed effective-allocation threshold $a_{\min}$, the effective active set is

(4) $$I_{t}\left(a_{\min}\right)=\left\{i:a_{t,i}\geq a_{\min}\right\}.$$

This set denotes regions that receive enough capacity to create an execution-layer scheduling effect. The environment transition is

(5) $$s_{t+1}=T\left(s_{t},a_{t},\xi_{t}\right),$$

where $\xi_{t}$ collects the traffic-arrival and disturbance draws. Graph construction and initialization/reset are specified in Appendix A.1 and Appendix A.2, respectively; Appendix A.3 gives the complete transition in Equations (A14)–(A29).

Normalized end-to-end emergency communication support capability is determined by current communication interruption, local access capability, upstream support capability, and traffic queue. The unclipped capability is

(6) $$\begin{array}{cc} {\tilde{C}}_{t} & =\frac{\sum_{i}v_{i}\left(1-g_{t,i}\right){\left(\eta_{t,i}^{\mathrm{loc}}\right)}^{1+\lambda_{\zeta }\zeta_{i}}\kappa_{t,i}^{\sup}{\left[1-\frac{Q_{t,i}}{{\bar{Q}}_{i}+\varepsilon }\right]}_{+}}{\sum_{i}v_{i}+\varepsilon },\\ C_{t} & =\min\left\{\max\left\{{\tilde{C}}_{t},0\right\},1\right\},\;C_{t}\in \left[0,1\right]. \end{array}$$

where ${\left[x\right]}_{+}=\max\left\{x,0\right\}$. The multiplicative form encodes a series-reliability assumption: an unavailable, locally unsupported, disconnected, or fully backlogged region contributes no usable planning capability, and partial factors reduce its contribution jointly rather than additively. The exponent $p_{i}=1+\lambda_{\zeta }\zeta_{i}$ applies a stronger penalty to a local-capability shortfall when the region has greater upstream dependency; $p_{i}=1$ recovers the unamplified product. Holding the other normalized factors fixed and taking $0<\eta_{t,i}^{\mathrm{loc}}\leq 1$,

$$\frac{\partial {\tilde{C}}_{t}}{\partial \lambda_{\zeta }}=\frac{\sum_{i}A_{t,i}\zeta_{i}{\left(\eta_{t,i}^{\mathrm{loc}}\right)}^{p_{i}}\log\eta_{t,i}^{\mathrm{loc}}}{\sum_{i}v_{i}+\varepsilon }\leq 0,\;A_{t,i}=v_{i}\left(1-g_{t,i}\right)\kappa_{t,i}^{\sup}{\left[1-\frac{Q_{t,i}}{{\bar{Q}}_{i}+\varepsilon }\right]}_{+},$$

with the zero-capability boundary defined by continuity. Thus $\lambda_{\zeta }$ monotonically increases the penalty attached to dependency-sensitive local shortfall. This is a bounded, interpretable state-evaluation proxy, not a physical throughput formula.

Equation (6) is a state-evaluation proxy rather than an action-dependent differentiable objective. Allocation affects the next capability through the transition, so the complete map $a_{t}\mapsto C_{t+1}$ is generally neither concave nor everywhere differentiable. The proxy is continuous on the compact normalized domain, but its gradient is not globally Lipschitz. PPO evaluates likelihood and entropy in the 15-dimensional control space and treats the decoder and simulator as part of the environment; gradients are not propagated through the capability proxy or simplex projection.

The communication-support loss depends on the critical-region condition and upstream route condition. The corresponding region sets are

$$K=\left\{i:\chi_{i}^{\mathrm{critical}}\geq 0.70\right\},\;T=\left\{i:{\mathrm{type}}_{i}\in \left\{\mathrm{airfield},\mathrm{assembly},\mathrm{transfer}\right\}\right\}.$$

K is determined by regional criticality, whereas T contains regions serving entry, remote-demand, or backhaul functions. Both sets remain fixed within an episode. Queue pressure uses the clipped ratio ${\bar{Q}}_{t,i}^{\mathrm{ratio}}=\min\left\{Q_{t,i}/\left({\bar{Q}}_{i}+\varepsilon \right),1\right\}$. The loss components are

(7) $$\begin{array}{cc} L_{t}^{\mathrm{critical}} & =\frac{\sum_{i\in K}\chi_{i}^{\mathrm{critical}}g_{t,i}}{\sum_{i\in K}\chi_{i}^{\mathrm{critical}}+\varepsilon },\\ L_{t}^{\mathrm{support}} & =1-\frac{\sum_{i\in T}v_{i}\kappa_{t,i}^{\sup}}{\sum_{i\in T}v_{i}+\varepsilon },\\ L_{t}^{\mathrm{backlog}} & =\frac{\sum_{i}v_{i}{\bar{Q}}_{t,i}^{\mathrm{ratio}}}{\sum_{i}v_{i}+\varepsilon },\;L_{t}^{e2e}=1-C_{t}. \end{array}$$

They represent critical-region access outage, upstream relay/backhaul path shortfall, traffic backlog, and end-to-end emergency communication recovery shortfall. The total loss is

(8) $$L_{t}=\omega_{1}L_{t}^{\mathrm{critical}}+\omega_{2}L_{t}^{\mathrm{support}}+\omega_{3}L_{t}^{\mathrm{backlog}}+\omega_{4}L_{t}^{e2e},$$

where the nonnegative weights $\omega_{1},\ldots ,\omega_{4}$ normalize different recovery-limiting signal channels into one planning-level communication-support objective and satisfy $\sum_{k}\omega_{k}=1$.

The one-step reward combines loss reduction, switching cost, and an optional effective-active penalty:

(9) $$R_{t}=L_{t}-L_{t+1}-\eta_{\mathrm{sw}}\frac{∥a_{t}-a_{t-1}{∥}_{1}}{B+\varepsilon }-\eta_{\mathrm{act}}\frac{1}{N}\sum_{i}I\left(a_{t,i}\geq a_{\min}\right),$$

where $\eta_{\mathrm{sw}}$ and $\eta_{\mathrm{act}}$ control capacity migration and effective activation, respectively, and $a_{\min}$ is the effective-allocation threshold. The loss terms, normalized switching distance, and active fraction are bounded dimensionless quantities, so Equation (9) is a weighted scalarization on normalized scales. A small switching weight is used to discourage unnecessary migration without imposing a hard reconfiguration limit; its sensitivity is evaluated in Section 5.2.

The finite-horizon policy objective is

(10) $$\begin{array}{cc} \max_{\pi_{\theta }}\; & J\left(\pi_{\theta }\right)=E_{\pi_{\theta }}\;\left[\sum_{t=0}^{H-1}\gamma_{\mathrm{RL}}^{t}R_{t}\right],\\ s.t.\; & u_{t}∼\pi_{\theta }\left(\cdot |s_{t}\right),\;a_{t}=D\left(s_{t},u_{t}\right)\;\mathrm{satisfies}\;\mathrm{Equation}\;(3). \end{array}$$

Here D denotes the deterministic decoder specified in Section 4, and $\gamma_{\mathrm{RL}}$ is the discount factor used by PPO; the subscript distinguishes it from the decoder score scale $\gamma_{t}$.

The resulting problem is to learn a control policy $\pi_{\theta }\left(u_{t}|s_{t}\right)$ whose deterministic decoder maps communication interruption, local access capability, upstream support, traffic queues, and end-to-end communication-support capability to regional allocations satisfying nonnegativity and total-capacity conservation. Positive-support and effective-activation behavior are evaluated separately.

## 4. Recovery-Limiting Signal-Conditioned Action Decoding

**Figure 2:**
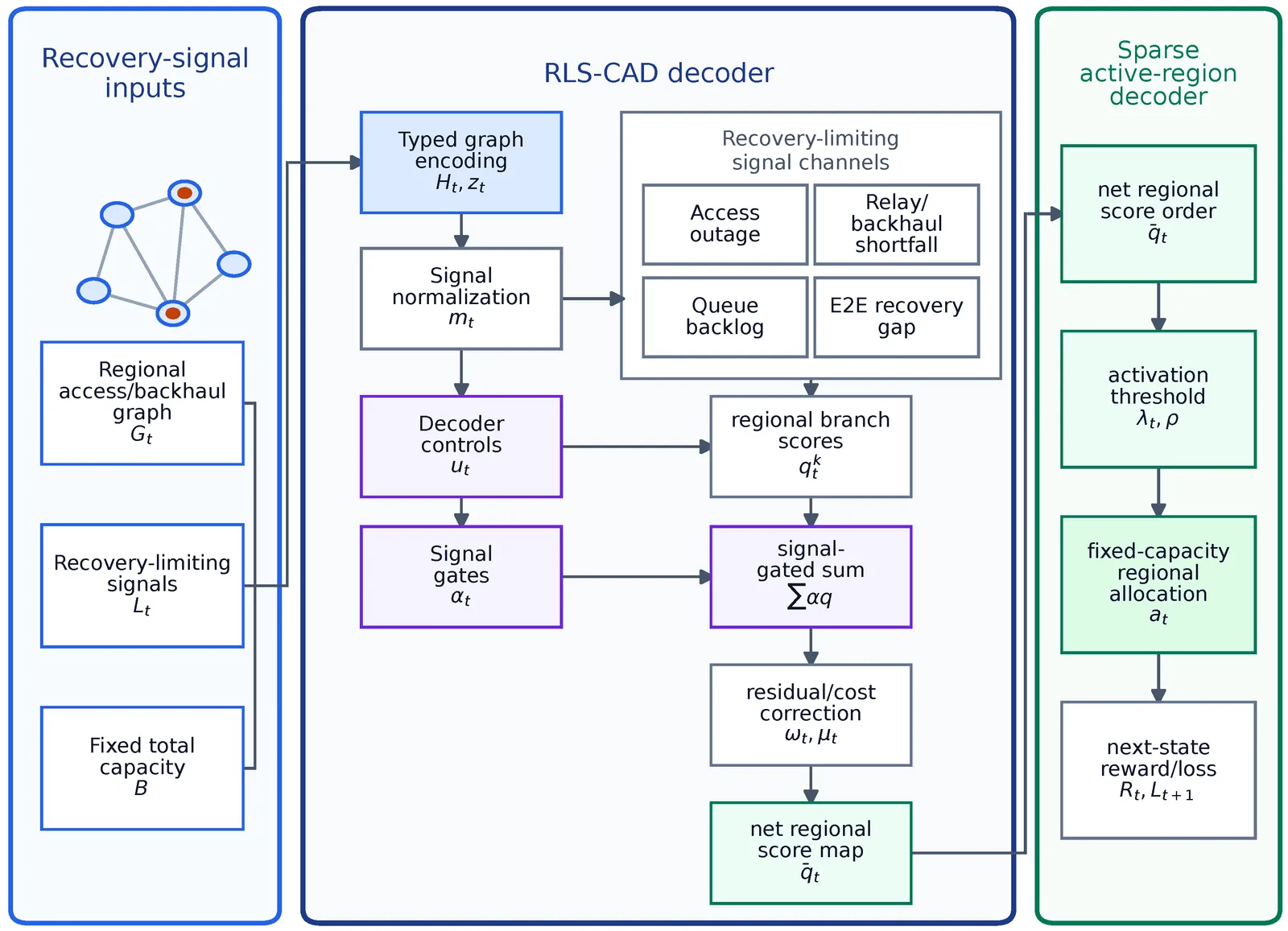
RLS-CAD framework. The regional access–backhaul graph, four observable recovery-limiting signal maps, and the fixed per-period capacity allocation first enter typed graph encoding and signal normalization. The normalized descriptor provides critical-region access outage, relay/backhaul shortfall, queue backlog, and end-to-end (E2E) recovery-gap channels. The policy actor controls signal gates, regional score shape, residual/cost correction, and projection variables. The decoder then fuses regional branch scores with residual correction and placement-cost adjustment, and activation-threshold simplex projection produces a nonnegative total-capacity-conserving regional allocation. The next-state reward/loss signal is returned to the policy-improvement interface.

RLS-CAD converts the observable access–backhaul state into a nonnegative fixed-total-capacity allocation while avoiding diffuse ineffective placement. Access outage, relay/backhaul shortfall, queue backlog, and end-to-end recovery gap must shape both support selection and allocation magnitude to guide action generation. These signals are normalized on the typed graph, after which the policy actor produces signal gates, regional-score corrections, and projection controls. The controls regulate score-based fusion, execution-cost correction, and the final support set. In Figure 2, the environment provides the regional graph, recovery indicators, and per-period capacity; the decoder generates a sparse regional action; and the next-state reward and loss support policy improvement.

### 4.1. Recovery-Limiting Signal Construction

Recovery-limiting signal construction uses four computable access–backhaul recovery constraints. Critical-region access outage measures interruption over the threshold-defined set K in Equation (7); it is broader than the displayed priority-access role alone. Upstream relay/backhaul shortfall measures the loss of support that can block downstream communication. End-to-end recovery shortfall measures the current inability of the access–backhaul chain to restore regional connectivity, and unserved traffic queue backlog measures accumulated demand that remains after current capacity support. These four channels are derived from the current planning-layer state, so each variable has a defined communication-support meaning before it enters the decoder.

The policy uses a 24-dimensional regional input, a 10-dimensional global input, and two typed message-passing layers. Exact inputs, adjacency preprocessing, layer widths, activations, output heads, and parameter count are specified in Appendix A.4, Equations (A30)–(A37), and Table A4.

The decoder constructs a four-component pressure descriptor. For channel k∈M={critical,support,backlog,e2e}, define $I_{k}=\max\left(L_{0}^{k},L_{\min}\right)$, $p_{k}=\omega_{k}/\left(\frac{1}{4}\sum_{h}\omega_{h}\right)$, and let $s_{k}>0$ denote the channel reference scale. For each component *x* below, $A_{x}\left(z\right)=\langle \beta_{x},z\rangle$ denotes a nonnegative linear aggregation. The relative, absolute, and worsening pressures are

(11) $$\begin{array}{cc} r_{t,k}^{\mathrm{rel}} & =p_{k}L_{t}^{k}/I_{k}, \end{array}$$

(12) $$\begin{array}{cc} r_{t,k}^{\mathrm{abs}} & =\sqrt{p_{k}\max\left(L_{t}^{k},0\right)/s_{k}}, \end{array}$$

(13) $$\begin{array}{cc} r_{t,k}^{\mathrm{wor}} & =p_{k}\max\left(L_{t}^{k}-L_{t-1}^{k},0\right)/I_{k}. \end{array}$$

Their combination gives the descriptor component

(14) $$m_{t,k}=\mathrm{clip}\;\left(A_{m}\left(\left[r_{t,k}^{\mathrm{rel}},r_{t,k}^{\mathrm{abs}},r_{t,k}^{\mathrm{wor}}\right]\right),0,m_{\max}\right).$$

At reset, $L_{-1}^{k}=L_{0}^{k}$, so the worsening term in Equation (13) is zero. Appendix A.4.2 and Table A2 give the fixed reference configuration used in the reported experiments.

### 4.2. Recovery-Limiting Signal-Conditioned Policy Controls

RLS-CAD samples a 15-dimensional diagonal-Gaussian control before deterministic decoding,

(15) $$u_{t}∼N\;\left(\mu_{\theta }\left(s_{t}\right),\mathrm{diag}\;\left(\exp(2l_{\sigma })\right)\right),\;u_{t}\in R^{15}.$$

For a sampled value $u_{t}$, PPO uses the summed Gaussian log-likelihood and entropy

(16) $$\begin{array}{cc} \log\pi_{\theta }\left(u_{t}|s_{t}\right) & =-\frac{1}{2}\sum_{j=1}^{15}\left[\frac{{\left(u_{t,j}-\mu_{\theta ,j}\right)}^{2}}{\exp(2l_{\sigma ,j})}+2l_{\sigma ,j}+\log\left(2\pi \right)\right], \end{array}$$

(17) $$\begin{array}{cc} H[\pi_{\theta }\left(\cdot |s_{t}\right)] & =\sum_{j=1}^{15}\left[l_{\sigma ,j}+\frac{1}{2}\log\left(2\pi e\right)\right]. \end{array}$$

These quantities are evaluated for the sampled, unclipped $u_{t}$. The environment then applies ${\tilde{u}}_{t}=\mathrm{clip}\left(u_{t},-8,8\right)$ coordinatewise; no separate likelihood or entropy is assigned to the decoded *N*-dimensional allocation.

Coordinates 1:4 control signal gating, coordinate 5 controls projection temperature, coordinates 6 and 7 control the score and execution-cost scales, coordinates 8:11 control channel gains, and coordinates 12:15 control residual corrections. Appendix A.4.3 and Table A3 give the exact transformations, constants, and induced ranges.

Let ${\hat{\omega }}_{k}=\log\left(\max\left(\omega_{k},\omega_{\min}\right)/\omega_{\mathrm{ref}}\right)$. The decoder gate is

(18) $$\alpha_{t}=\mathrm{sparsemax}\;\left(\lambda_{u}{\tilde{u}}_{t,1:4}+\lambda_{m}Mm_{t}+\lambda_{\omega }\hat{\omega }\right).$$

Sparsemax [14] gives $\alpha_{t,k}\geq 0$ and $\sum_{k}\alpha_{t,k}=1$. Its controlled comparison with a temperature-scaled softmax gate is reported in Section 5.4.

For the four score maps, let $r_{t,i}^{Q}$ be defined as above, $p_{i}=1+\lambda_{\zeta }\zeta_{i}$, and $U{\left(x\right)}_{i}={\left[x_{i}\right]}_{+}/\left(\max_{j}{\left[x_{j}\right]}_{+}+\varepsilon \right)$. The target-side capability sensitivity is

(19) $$c_{t,j}=v_{j}\left(1-g_{t,j}\right)\kappa_{t,j}^{\sup}\left(1-r_{t,j}^{Q}\right)p_{j}\max{\left(\eta_{t,j}^{\mathrm{loc}},\varepsilon \right)}^{p_{j}-1}.$$

Aggregating this sensitivity over the outgoing support edges of provider *i* gives

(20) $$d_{t,i}^{\mathrm{down}}=\sum_{j}A_{ij}^{\sup}\frac{\zeta_{j}c_{t,j}}{\sum_{h}A_{hj}^{\sup}+\varepsilon }.$$

The provider response ratio and its current recovery gap are

(21) $$\begin{array}{cc} r_{t,i}^{\mathrm{rep}} & =\frac{e_{i}^{\mathrm{rep}}\left(1+r_{t,i}^{\mathrm{ready}}\right)}{2(s_{i}^{\mathrm{rep}}+\varepsilon )}, \end{array}$$

(22) $$\begin{array}{cc} h_{t,i}^{\mathrm{gap}} & =A_{\mathrm{gap}}\left(\left[g_{t,i},r_{t,i}^{Q},1-r_{t,i}^{\mathrm{ready}},1-\eta_{t,i}^{\mathrm{loc}}\right]\right). \end{array}$$

Their product defines the local provider-marginal proxy

(23) $$\Delta C_{t,i}^{\mathrm{loc}}=d_{t,i}^{\mathrm{down}}r_{t,i}^{\mathrm{rep}}h_{t,i}^{\mathrm{gap}}.$$

Thus, Equation (23) is a deterministic recovery proxy rather than a numerical finite difference.

The route-break ratio is

$$b_{t,i}^{\mathrm{break}}=\frac{\sum_{j}A_{ij}^{\mathrm{route}}\left(1-h_{t,ij}^{\mathrm{route}}\right)}{\max(\sum_{j}A_{ij}^{\mathrm{route}},1)}.$$

Define the support and backlog feature vectors as

$$z_{t,i}^{\sup}=\left[\left(1-\kappa_{t,i}^{\sup}\right)v_{i},b_{t,i}^{\mathrm{break}},b_{i}^{\mathrm{corr}}\right],\;z_{t,i}^{\mathrm{back}}=\left[r_{t,i}^{Q},1-r_{t,i}^{\mathrm{ready}},d_{t,i}^{\mathrm{demand}}\right].$$

The critical, support, and backlog maps are

(24) $$\begin{array}{cc} q_{t,i}^{\mathrm{critical}} & =\chi_{i}v_{i}g_{t,i}A_{\mathrm{critical}}\left(\left[1,\eta_{t,i}^{\mathrm{loc}}\right]\right), \end{array}$$

(25) $$\begin{array}{cc} q_{t,i}^{\mathrm{support}} & =A_{\sup}\left(z_{t,i}^{\sup}\right), \end{array}$$

(26) $$\begin{array}{cc} q_{t,i}^{\mathrm{backlog}} & =A_{\mathrm{back}}\left(z_{t,i}^{\mathrm{back}}\right). \end{array}$$

The end-to-end map first constructs bridge, chain-gap, and anchor components:

(27) $$\begin{array}{cc} b_{t,i}^{\mathrm{bridge}} & =b_{i}^{\mathrm{corr}}b_{t,i}^{\mathrm{break}}\left(1-\kappa_{t,i}^{\sup}\right)A_{\mathrm{bridge}}\left(\left[1,b_{i}^{\mathrm{route}}\right]\right), \end{array}$$

(28) $$\begin{array}{cc} h_{t,i}^{\mathrm{chain}} & =v_{i}\zeta_{i}\left\{1-\left(1-g_{t,i}\right)\eta_{t,i}^{\mathrm{loc}}\kappa_{t,i}^{\sup}\left(1-r_{t,i}^{Q}\right)\right\}r_{t,i}^{\mathrm{rep}}, \end{array}$$

(29) $$\begin{array}{cc} a_{t,i}^{\mathrm{anchor}} & =A_{\mathrm{anchor}}\left(\left[q_{t,i}^{\mathrm{critical}},q_{t,i}^{\mathrm{support}},q_{t,i}^{\mathrm{backlog}}\right]\right). \end{array}$$

Their normalized combination gives

(30) $$q_{t,i}^{e2e}=A_{e2e}\left(\left[U{\left(\Delta C_{t}^{\mathrm{loc}}\right)}_{i},U{\left(b_{t}^{\mathrm{bridge}}\right)}_{i},U{\left(h_{t}^{\mathrm{chain}}\right)}_{i},U{\left(a_{t}^{\mathrm{anchor}}\right)}_{i}\right]\right).$$

The four maps in Equations (24)–(30) enter the standardization in Equation (31); U is applied only to the four nonnegative components of Equation (30). Appendix A.1 defines $b_{i}^{\mathrm{corr}}$ as a normalized corridor-centrality proxy. All four maps are deterministic functions of the current state and graph.

### 4.3. Finite-Support Capacity-Allocation Decoding

Let $S{\left(x\right)}_{i}=\left(x_{i}-{\mathrm{mean}}_{j}x_{j}\right)/\left({\mathrm{std}}_{j}x_{j}+10^{-6}\right)$, where std is the population standard deviation over regions. The fused score is

(31) $$q_{t}\left(i\right)=\gamma_{t}\sum_{k\in M}\alpha_{t,k}\nu_{t,k}S{\left(q_{t}^{k}\right)}_{i}+\gamma_{t}\sum_{k\in M}\varrho_{t,k}b_{t}^{k}\left(i\right),$$

where the four residual bases $b_{t}^{k}$ are, in order, $S(g_{t})$, $S(1-\kappa_{t}^{\sup})$, $S(r_{t}^{Q})$, and $S(1-\eta_{t}^{\mathrm{loc}})$. Here $\varrho_{t,k}$ are the residual coefficients in Table A3, whereas $\omega_{k}$ are the objective weights. The execution-aware net score is

(32) $${\bar{q}}_{t}\left(i\right)=q_{t}\left(i\right)-\mu_{t}c_{i}^{\mathrm{exec}}.$$

The static normalized placement-cost proxy is

(33) $$c_{i}^{\mathrm{exec}}=\mathrm{clip}\;\left(A_{c}\left(\left[1,d_{i}^{c},1-b_{i}^{\mathrm{route}}\right]\right),c_{\min},c_{\max}\right).$$

Here $d_{i}^{c}$ is normalized distance from the map center and $b_{i}^{\mathrm{route}}$ is normalized route-incidence strength. Equation (33) represents spatial placement difficulty and route accessibility; dynamic switching is penalized separately in Equation (9).

Allocation-threshold projection converts net scores into regional capacity-allocation actions and selects their positive support. The positive-support size and allocation threshold are determined jointly from the current net-score distribution, execution-cost penalty, and projection temperature; no fixed support size is prescribed. Given ${\bar{q}}_{t}$, allocation weights are obtained by

(34) $$w_{t}=\arg\max_{w\in \Delta^{N-1}}\left({\bar{q}}_{t}^{⊤}w-\frac{\tau_{t}}{2}{∥w∥}_{2}^{2}\right),\;a_{t}=Bw_{t}.$$

The closed form is

(35) $$w_{t,i}=\max\left(\frac{{\bar{q}}_{t}\left(i\right)-\lambda_{t}}{\tau_{t}},0\right),\;\sum_{i}w_{t,i}=1,$$

where $\lambda_{t}$ is the relative allocation threshold induced by the simplex constraint and changes with the current net-score distribution and projection temperature. Only regions with net scores above this scenario-dependent threshold receive positive allocation. Let ${\bar{q}}_{t,(1)}\geq \cdots \geq {\bar{q}}_{t,(N)}$ be sorted net scores and $P_{k}=\sum_{j=1}^{k}{\bar{q}}_{t,(j)}$ be the sorted prefix sum. The positive-support size is

(36) $$\rho =\max\left\{k:{\bar{q}}_{t,(k)}>\frac{P_{k}-\tau_{t}}{k}\right\},\;\lambda_{t}=\frac{P_{\rho }-\tau_{t}}{\rho }.$$

The positive-support set is therefore determined by the state-conditioned scores, execution cost, and projection temperature. Equation (34) is the Euclidean projection $w\left(\bar{q}\right)=\Pi_{\Delta^{N-1}}\left(\bar{q}/\tau \right)$. Its strongly concave objective has a unique solution for every τ>0, and sorting with prefix sums returns that solution in finite operations. Non-expansiveness gives

$$∥w\left(\bar{q}\right)-w\left({\bar{q}}^{\prime }\right){∥}_{2}\leq \frac{1}{\tau }{∥\bar{q}-{\bar{q}}^{\prime }∥}_{2}.$$

When ρ changes, an entering or leaving coordinate is zero at the boundary, so *w* remains continuous. The deterministic decoder computes ρ and $\lambda_{t}$ outside PPO backpropagation. These properties establish uniqueness and stability of each projection call, not global convergence of PPO training. The projection guarantees nonnegativity, total-capacity conservation, and exact zeros, but it does not ensure $a_{t,i}\geq a_{\min}$ for every positive component. We therefore distinguish the positive support $P_{t}=\left\{i:a_{t,i}>\varepsilon_{\mathrm{num}}\right\}$ from the effective active set $I_{t}=\left\{i:a_{t,i}\geq a_{\min}\right\}$; $a_{\min}$ is used only as an effectiveness threshold. Subthreshold capacity and support changes are evaluated in Section 5.4. With a fixed number of signal channels, score construction and fusion cost O(N), sparse support aggregation costs $O(|E^{\sup}|)$, and the sorting-based projection costs O(NlogN). The decoder therefore has complexity $O(|E^{\sup}|+N\log N)$ and O(N) auxiliary memory. Measured inference latency and scaling are reported in Section 5.6.

### 4.4. Algorithmic Summary

The preceding modules form a complete action-generation procedure. The policy actor reads the typed graph representation and recovery-limiting signals, outputs compact decoder controls, and the structured decoder deterministically converts those controls into a feasible regional capacity-allocation action. A critic or value estimator can evaluate its long-term effect and provide policy-improvement signals. Algorithm 1 summarizes one decision period. It constructs the recovery signals, obtains decoder controls, forms regional scores, decodes the allocation, and returns the next-state reward and losses.

**Algorithm 1** Recovery-Limiting Signal-Conditioned Action Decoding (RLS-CAD)**Input:** Regional graph state $s_{t}$, communication-support loss channels ${\left\{L_{t}^{k}\right\}}_{k\in M}$, total capacity *B*, effective-allocation threshold $a_{\min}$, and typed adjacency matrices $\left\{A^{r}\right\}$.
**Output:** Feasible allocation action $a_{t}$, effective active set $I_{t}\left(a_{\min}\right)$, decoder variables $\alpha_{t},\lambda_{t}$, next state $s_{t+1}$, losses $\left\{L_{t+1}^{k}\right\}$, and reward $R_{t}$.
  1:Build node features $h_{t,i}^{\left(0\right)}$ from regional attributes, current communication states, and the previous allocation.  2:Encode spatial, route, and symmetrized support edges to obtain $H_{t}$ and $z_{t}$ using the architecture in Appendix A.4.  3:Construct the four-component recovery-pressure descriptor $m_{t}$ by Equations (11)–(14).  4:Sample $u_{t}$ by Equation (15), retain its Gaussian log-likelihood for PPO, and clip only the decoder input to ${\left[-8,8\right]}^{15}$.  5:Map all 15 coordinates by Table A3 and compute $\alpha_{t}$ using the decoder gate in Equation (18).  6:Construct $\Delta C_{t}^{\mathrm{loc}}$ and the four regional score maps by Equations (19)–(23) and (24)–(30).  7:Fuse standardized maps using $\alpha_{t}$, $\nu_{t}$, $\gamma_{t}$, and $\varrho_{t}$.  8:Apply execution-cost correction to obtain net regional score ${\bar{q}}_{t}$ by Equations (31), (32), and (33).  9:Sort ${\bar{q}}_{t}$, compute prefix sums $P_{k}$, and obtain ρ and $\lambda_{t}$ by Equation (36).10:Project onto the capacity simplex with $w_{t,i}=\max\left(\left({\bar{q}}_{t}\left(i\right)-\lambda_{t}\right)/\tau_{t},0\right)$ and set $a_{t}=Bw_{t}$.11:Return $I_{t}\left(a_{\min}\right)$, execute $a_{t}$, and output $s_{t+1}$, $\left\{L_{t+1}^{k}\right\}$, and $R_{t}$ for policy evaluation.


## 5. Experiments

The experiments evaluate fixed-total-capacity allocation under common graph-generation and transition rules. We first compare overall recovery performance and then examine fair controls, key-parameter sensitivity, and structural transfer.

### 5.1. Experimental Setup

The main experiment uses a 12×15 planning-level graph (N=180), horizon H=40, and normalized capacity B=1. Five reset profiles model key-region damage, route fracture, backlog, distributed degradation, and their compound; the 100 fixed evaluation seeds contain 20 scenarios of each profile. The main transition is deterministic after the seeded reset. Role and edge rules are given in Appendix A.1; profile distributions, loss weights, and the reset procedure in Appendix A.2 and Table A1; and the transition equations in Appendix A.3. Link-level execution is abstracted as regional quotas, so FinalCap and TaskUtility are normalized planning metrics rather than physical throughput. Every method uses the same environment and scenario states.

Evaluation covers recovery performance, action sparsity, and reconfiguration cost. FinalCap measures terminal recovery, CTI measures capability gain, TaskUtility measures terminal task utility, and EpisodeReturn is the cumulative return. EffectiveActiveRatio and ExactZeroRatio describe effective placement and exact-zero allocation, while SwitchCost and SubthresholdMass describe capacity migration and capacity below the effective threshold. Graph-PPO is the main graph-based learning baseline [11,30]. Structural variants replace the gate or budget map and remove selected branches. Both information-matched controls receive the same recovery signals, marginal-capability feature, and execution cost, and their actors directly output *N* regional scores. They omit the RLS-CAD gate, residual correction, and projection controls and use either Softmax or direct sparse projection [13,14]. Rule-based comparisons cover uniform, priority-based, and recovery-greedy allocation. Every method outputs regional actions under the same constraints.

Each learning method is trained for 80,000 environment steps with seeds 0:4 and the final checkpoint rule [3]. PPO uses learning rate $2.5\times 10^{-4}$, rollout length 256, batch size 128, discount 0.99, GAE parameter 0.95, clipping range 0.20, 10 optimization epochs per rollout, value-loss coefficient 0.5, and maximum gradient norm 0.5. The entropy coefficient is 0.001 for the 15-dimensional reference action; direct 180-dimensional controls use $0.001\left(15/180\right)=8.3333\times 10^{-5}$ so the summed-entropy contribution is dimension matched. For the main comparison and profile table, crossed training-seed–scenario bootstrap intervals use 20,000 replicates; paired Wilcoxon tests operate on the 100 scenario means, with Holm correction within each metric family. Exact analysis seeds and auxiliary settings are reported in Appendix A.5, and the software environment is listed in Table A5.

### 5.2. Access–Backhaul Recovery Performance

We first examine training stability by comparing RLS-CAD with five trainable baselines. The RLS-CAD trajectory aggregates five independent training seeds. Formal performance comparisons use 100 shared test scenarios; controlled variants are examined separately in Section 5.4.

**Figure 3:**
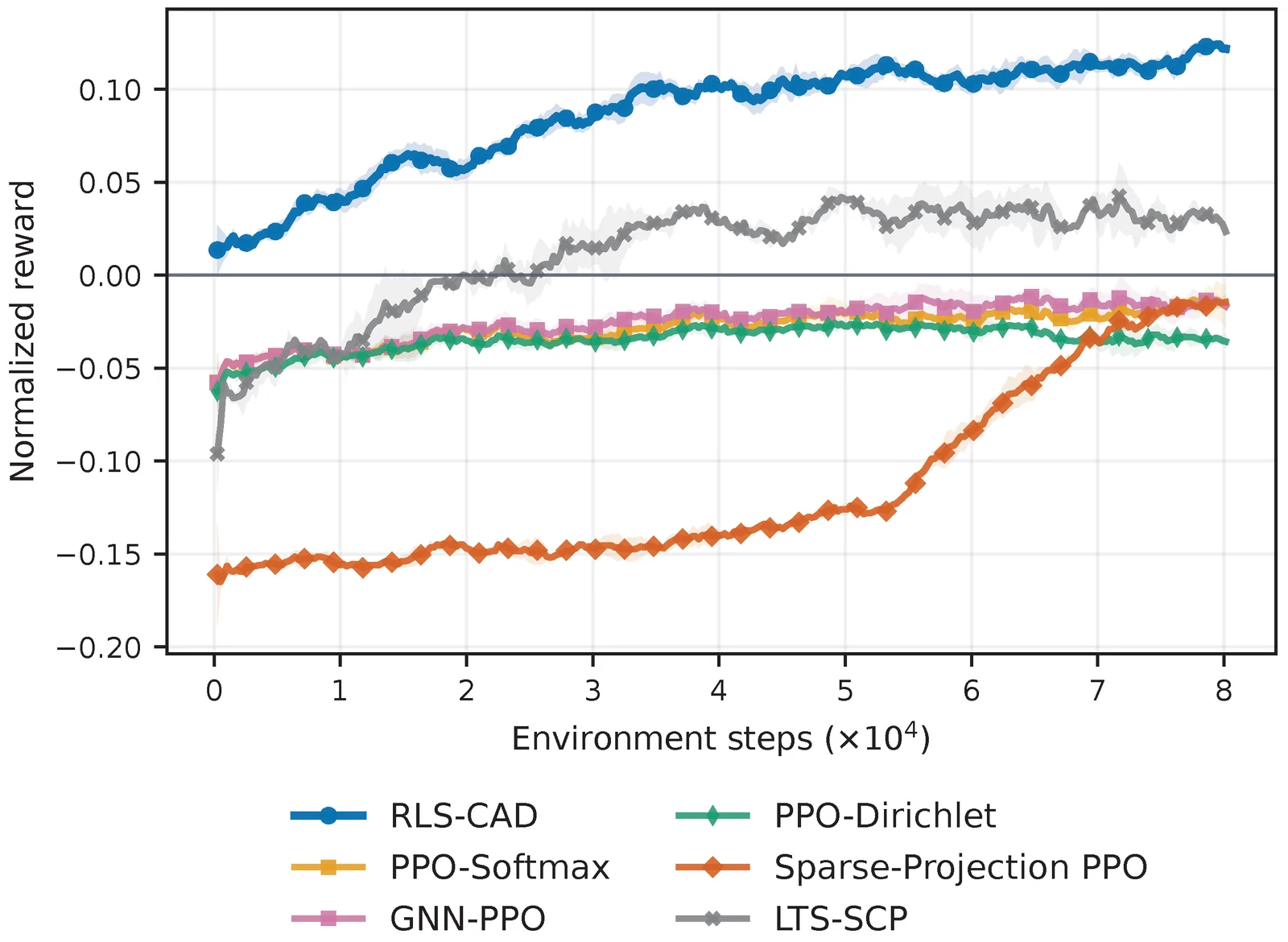
Normalized training-reward trajectories of RLS-CAD and five trainable baselines. The RLS-CAD curve aggregates five independent training seeds. Lines show seed means and shading denotes one seed-level standard error. Consecutive logged points are descriptive and are not treated as independent statistical samples.

The RLS-CAD trajectory remains stable and reaches the highest late-training reward range in Figure 3. This plot is descriptive because adjacent log points are temporally correlated. Performance is evaluated on 100 shared test scenarios. Table 1 reports terminal metrics and crossed intervals, while Table 2 and Table 3 provide individual-seed results and statistical comparisons.

**Figure 4:**
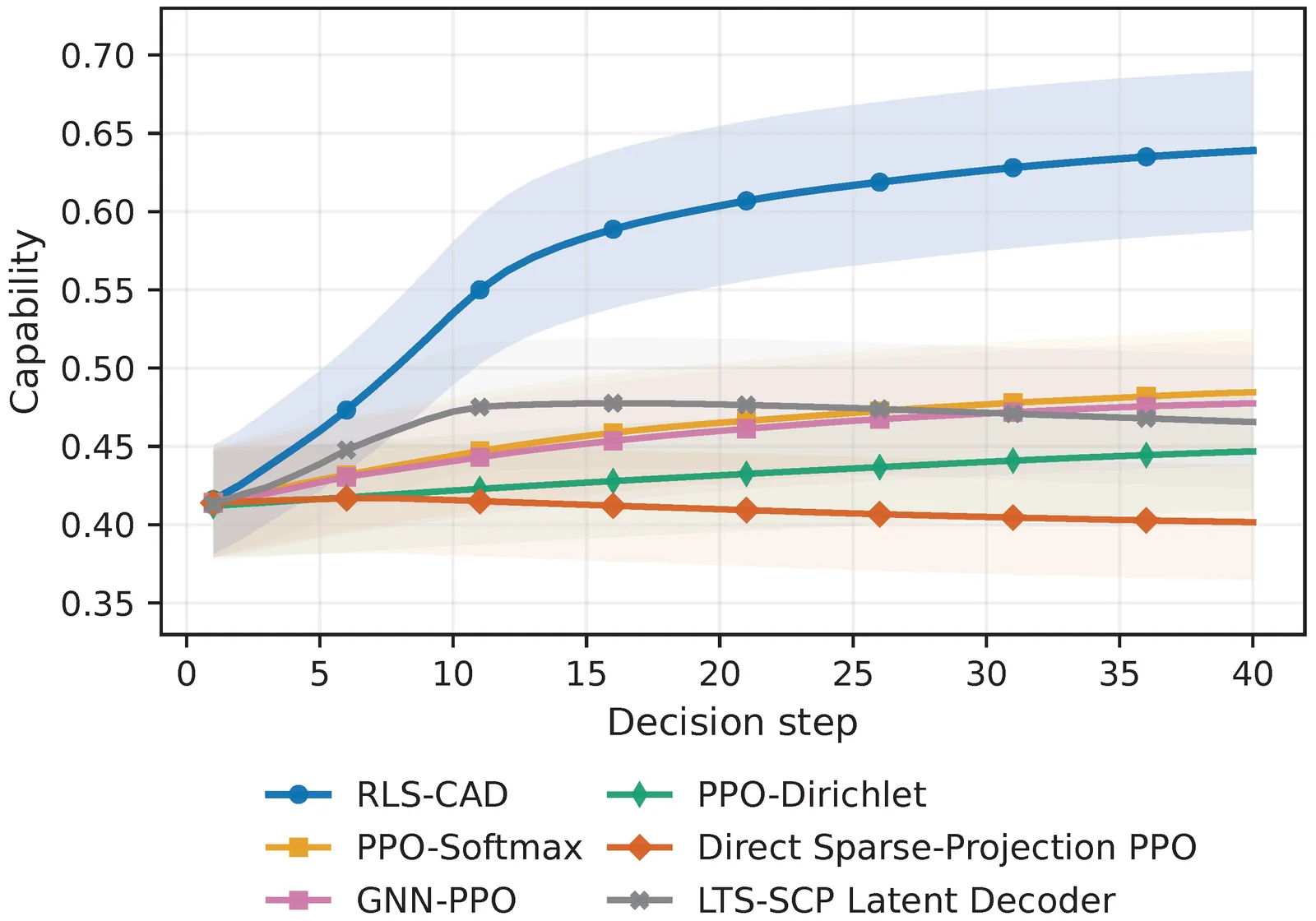
Decision-step emergency communication-support capability trajectories for learning and structured-decoder methods over the same 20 regional-graph test scenarios. Curves show scenario-level means and shaded bands show scenario-level standard errors.

**Figure 5:**
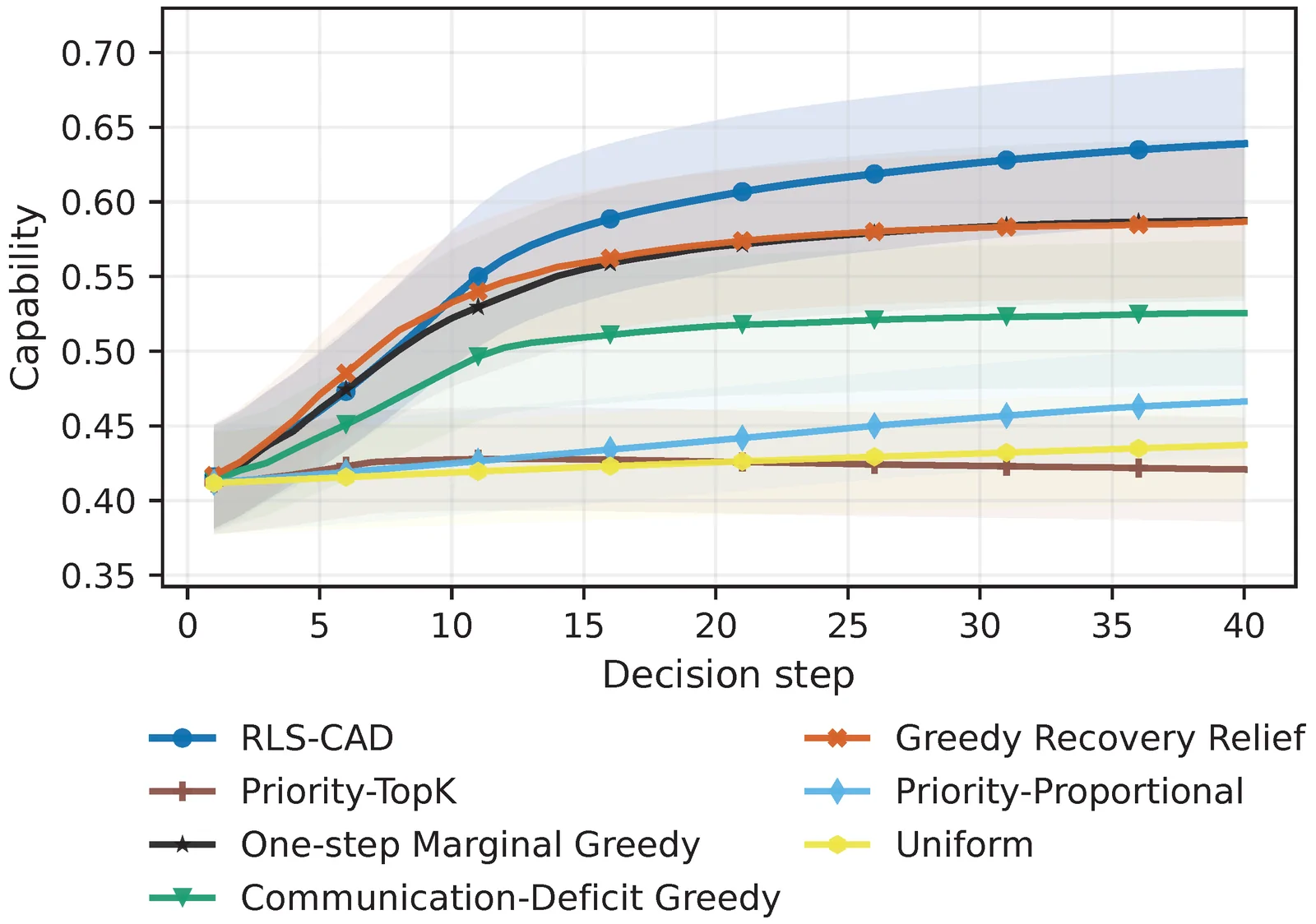
Decision-step emergency communication-support capability trajectories for rule-based and heuristic allocations over the same 20 regional-graph test scenarios. Curves show scenario-level means and shaded bands show scenario-level standard errors; RLS-CAD serves as the learned structured-decoder reference.

Decision-step capability trajectories show how action interfaces affect recovery over multiple periods. RLS-CAD restores end-to-end emergency communication-support capability faster on average and remains above PPO-Softmax, GNN-PPO, PPO-Dirichlet, Direct Sparse-Projection PPO, and the LTS-SCP-style Latent Decoder in later steps in Figure 4. One-step Marginal Greedy, Greedy Recovery Relief, and Communication-Deficit Greedy also improve communication-support capability in Figure 5, but their trajectories rely on explicit deficit ranking or one-step model evaluation and change more abruptly. Direct Sparse-Projection PPO produces highly concentrated actions but weaker communication-support capability, while Priority-TopK, Priority-Proportional, and Uniform improve more slowly.

Across terminal capability, capability gain, task utility, and episodic return, RLS-CAD significantly outperforms Graph-PPO. It also exceeds the external one-step marginal-greedy baseline, as summarized in Table 1.

#### 5.2.1. Switching-Cost and Mean-Preserving Arrival Sensitivity

We first test whether switching regularization restricts rapid emergency reconfiguration by training policies at three $\eta_{\mathrm{sw}}$ values and comparing terminal recovery, early capability gain, and capacity movement. We then increase arrival variability while preserving its mean to assess the sensitivity of four core policies to stochastic traffic fluctuations.

**Figure 6:**
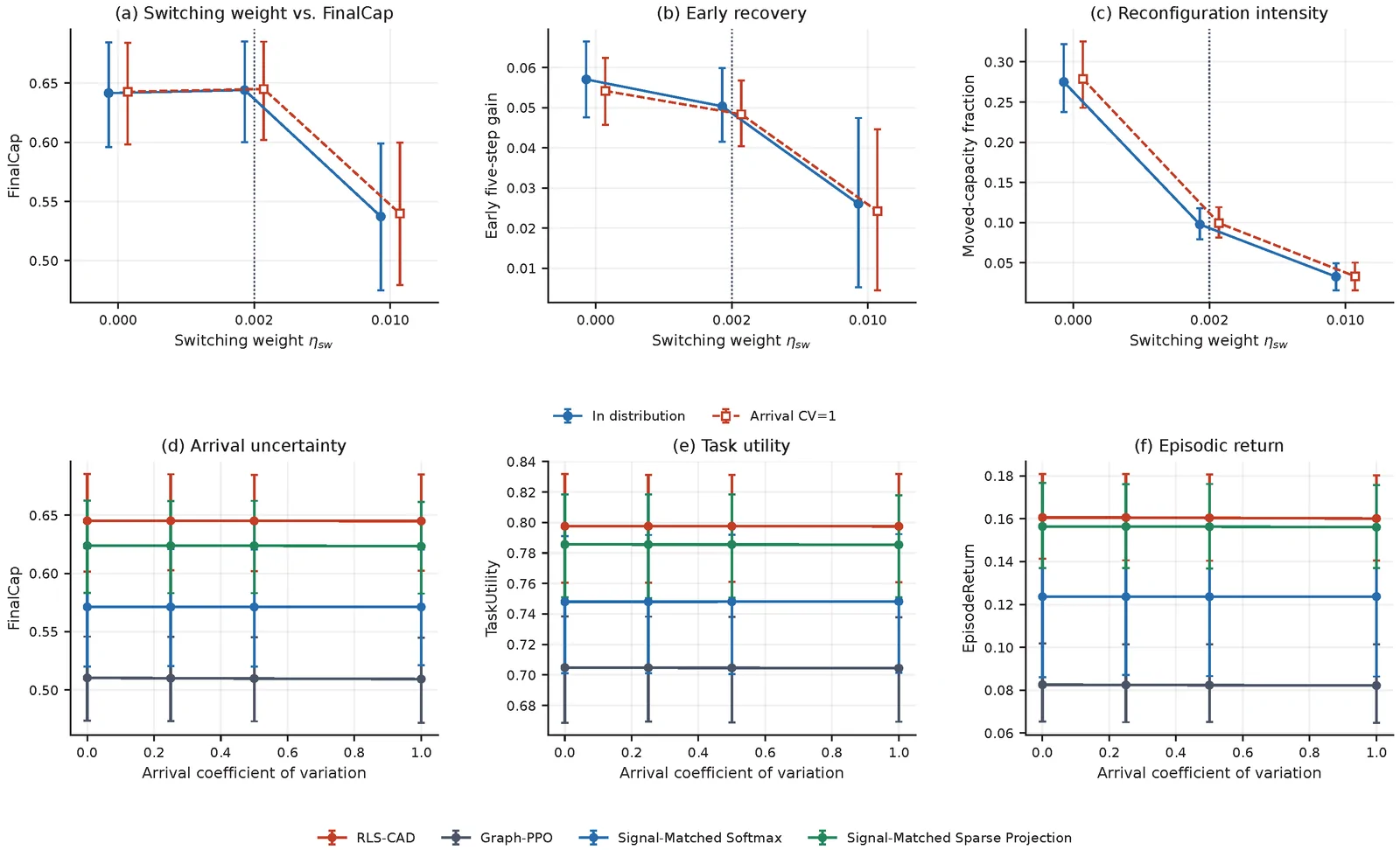
Switching-cost and arrival-noise sensitivity. Panels (**a**–**c**) compare final capability, early recovery, and capacity movement under three switching weights. In-distribution and arrival-CV = 1 estimates are horizontally offset around each weight for visibility; the vertical dotted line marks the default. Panels (**d**–**f**) compare four policies under mean-preserving arrival noise. Error bars are 95% crossed-bootstrap CIs.

The default $\eta_{\mathrm{sw}}=0.002$ provides a practical balance between recovery and migration, as shown in Figure 6a–c. Mean FinalCap is 0.644 and the moved-capacity fraction is 0.0976. Removing the penalty substantially increases migration without a resolved FinalCap improvement, whereas increasing it to 0.01 reduces migration but suppresses terminal and early recovery. Repeating the sweep at arrival CV 1 preserves this ordering; relative to the in-distribution condition, the absolute differences do not exceed 0.00249 for FinalCap or 0.00378 for moved-capacity fraction. Mean-preserving arrival noise causes only small changes for the four frozen policies, with every crossed interval including zero in Figure 6d–f. This finding applies to independent mean-preserving arrivals rather than persistent bursts or correlated link degradation.

#### 5.2.2. Decoder Sensitivity and Structural Transfer

To assess parameter robustness and the operating limits of the fixed-dimensional policy, we vary the dependency coefficient and projection temperature before evaluating the frozen policy under irregular topology, role relocation, and missing support edges. A separate cross-size test applies the same 15-dimensional policy to different region counts without further training.

**Figure 7:**
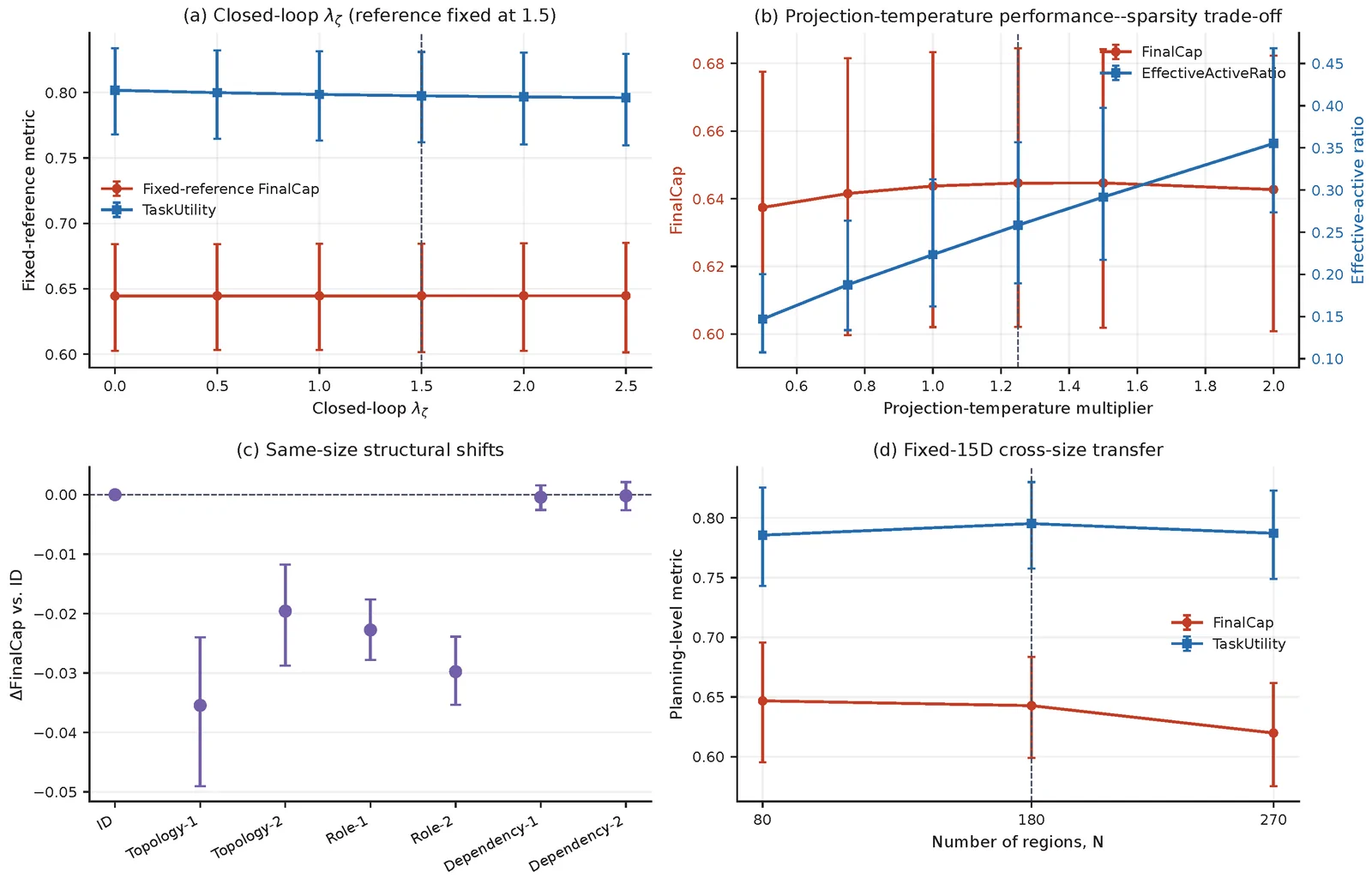
Decoder sensitivity and structural transfer. (**a**) Dependency-coefficient sensitivity; (**b**) performance and sparsity under projection-temperature changes; (**c**) FinalCap changes under three same-size structural shifts; (**d**) fixed-policy transfer across region counts. Error bars are 95% crossed-bootstrap CIs; dashed lines mark the defaults.

FinalCap remains nearly constant as the dependency coefficient changes, while TaskUtility declines only mildly, indicating that the coefficient primarily adjusts dependency sensitivity; this behavior appears in Figure 7a. Increasing projection temperature raises EffectiveActiveRatio, whereas FinalCap remains favorable near the default, making that value a performance–sparsity operating point. The structural and cross-size tests identify the generalization boundary: support-edge perturbation has little effect, irregular topology and role relocation cause clearer degradation, and performance decreases at N=270, as shown in Figure 7c,d. A control dimension independent of map size therefore does not imply size- or structure-invariant performance.

### 5.3. Spatial Capacity-Allocation Decoding Visualization

**Figure 8:**
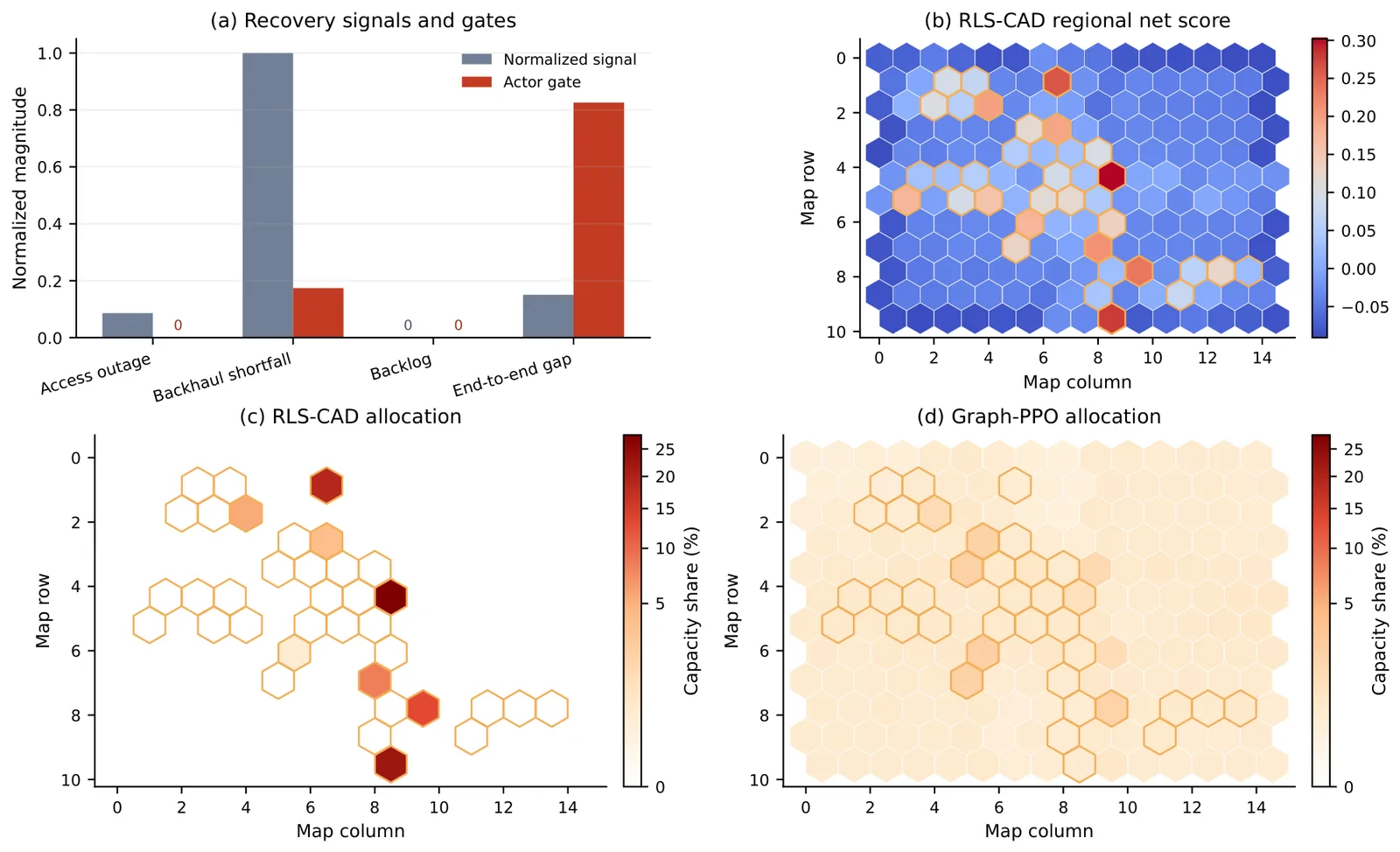
First decoded actions from the same initial backhaul-bottleneck state, where backlog has not yet accumulated. (**a**) Recovery signals and gates, with zero-height bars labeled 0; (**b**) RLS-CAD net scores; (**c**) RLS-CAD allocation; and (**d**) Graph-PPO allocation. The two policies produce 8 and 180 positive regions, respectively. Panels (**c**,**d**) use the same nonlinear scale to reveal small allocations; white denotes zero.

To examine how structured decoding changes the spatial distribution of capacity, we compare the first actions of RLS-CAD and Graph-PPO from the same backhaul-bottleneck state, before their trajectories diverge. Figure 8 presents the recovery signals, regional scores, and final allocations together to connect score formation with spatial placement.

In this example, the end-to-end and upstream-support signals raise scores near the critical path. RLS-CAD concentrates capacity on eight connected regions and assigns zero elsewhere. Graph-PPO assigns positive capacity to all 180 regions, although most shares are small and therefore appear in light colors. This example explains the spatial difference between the decoders; aggregate performance is assessed in Table 1.

### 5.4. Matched Controls and Projection Stability

To separate structural design from additional task information, we compare the complete decoder with two groups of controlled methods under the same reward, training budget, and test scenarios. The information-matched controls receive the same analytical inputs but directly map regional scores through Softmax or sparse projection. The internal variants replace the gate or allocation map, remove adaptive gating, or remove the end-to-end branch. RLS-CAD (Softmax Gate) changes only the Sparsemax branch gate to a temperature-controlled Softmax. Additional tests examine gate temperature and allocation behavior near the projection threshold.

**Figure 9:**
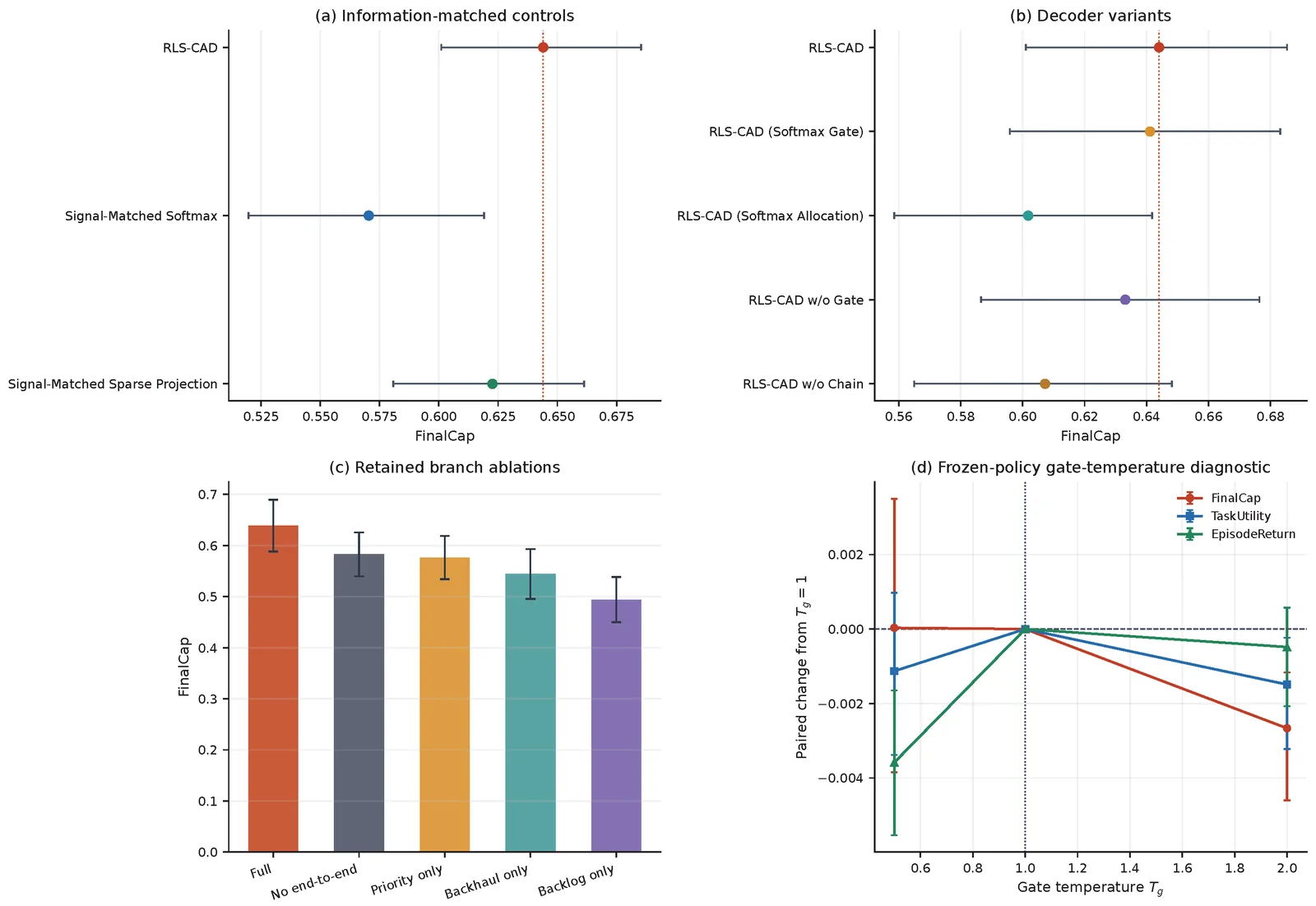
Controlled decoder comparisons and gate diagnosis. (**a**) FinalCap for the two information-matched controls; (**b**) FinalCap for the gate, allocation, and branch variants; (**c**) branch ablations reported as means with standard-error bars over 20 scenarios; and (**d**) paired changes produced by varying the Softmax-gate temperature of a frozen policy from $T_{g}=1$. In (**a**,**b**), point colors distinguish the methods and variants named on the vertical axes, filled points are overall means, horizontal lines are 95% crossed-bootstrap CIs, and the red dotted line marks the RLS-CAD mean.

With task information held constant, RLS-CAD remains better than both direct-allocation controls on FinalCap, CTI, and TaskUtility, as shown in Figure 9a and Table 3. Its EpisodeReturn difference from Signal-Matched Sparse Projection is small and its interval includes zero. Table 2 reports each learning method by seed. The structural variants in Figure 9b and the branch ablations in Figure 9c identify the effects of changing individual decoder components without treating them as external baselines. Gate-temperature changes do not produce a stable performance ordering, so we make no general superiority claim for Sparsemax over temperature-controlled Softmax.

**Figure 10:**
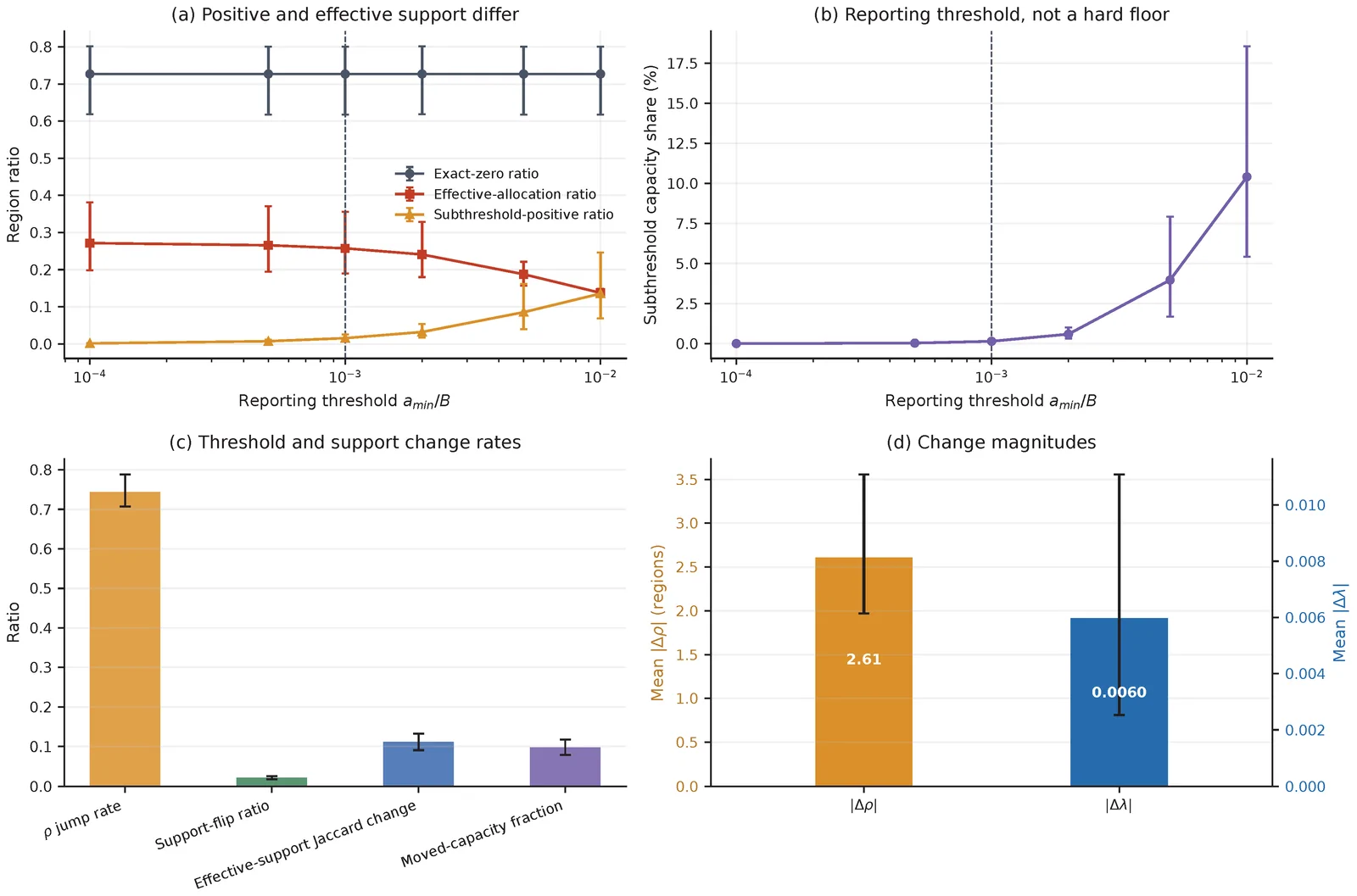
Allocation-threshold and projection-stability results for 5×100×40 RLS-CAD actions. (**a**) ExactZeroRatio, EffectiveActiveRatio, and the positive-but-subthreshold region ratio at each reporting threshold. (**b**) Capacity share below each threshold; $a_{\min}$ is a reporting threshold rather than a hard floor. (**c**) ρ-jump rate, support-flip ratio, effective-support Jaccard change, and moved-capacity fraction. (**d**) Mean absolute changes in ρ and $\lambda_{t}$, shown on separate left and right axes. Values inside the bars give the corresponding means, so the two bar heights are not directly comparable. Bars and curves show means with 95% crossed training-seed–scenario bootstrap intervals. Dashed lines mark $a_{\min}=10^{-3}B$.

The complete action analysis shows that only a small capacity share lies in $0<a_{t,i}<a_{\min}$. Although the support size changes, the affected regions remain localized between adjacent decisions, as summarized in Figure 10.

### 5.5. Performance by Disturbance Case

To determine whether the observed advantage depends on one disturbance type, this section evaluates every method separately on five access–backhaul disturbance profiles. Each profile contains 20 shared scenarios, allowing local damage, link failure, traffic backlog, and compound disruption to be compared under common conditions. The complete matrix includes both terminal capability and episodic return.

RLS-CAD retains its overall advantage over Graph-PPO across all five disturbance profiles, as reported in Table 4, showing that the improvement is not driven by one scenario type. Compound disruption is more difficult for all methods, and the best method varies with the metric. The profile-level comparison therefore supports stability across disturbance types but not universal superiority over every controlled decoder.

### 5.6. Planning-Inference Runtime and Scaling

To assess whether the decoder can support online decisions within a planning cycle, we measure single-sample inference latency under different hardware conditions and examine latency and memory as the number of regions grows. The protocol includes warm-up and repeated timing, and the cross-size test uses one fixed RLS-CAD checkpoint.

At N=180, complete inference for all learning methods is in the millisecond range, and projection contributes only a small share of the cost; Table 5 summarizes the measurements. RLS-CAD has lower latency on the measured CPU path, whereas Graph-PPO is faster when the actor is placed on the GPU. RLS-CAD latency and storage increase smoothly with the number of regions. These measurements support planning-cycle inference but do not constitute end-to-end physical-frame validation.

## 6. Conclusions

This paper proposes RLS-CAD for emergency wireless access–backhaul recovery. The method uses access outage, upstream shortfall, traffic backlog, and end-to-end recovery deficit to construct regional scores, and then generates fixed-total-capacity allocations through policy-controlled finite-support projection. Results from five training seeds and 100 shared test scenarios show that RLS-CAD significantly outperforms Graph-PPO. Controlled ablations, parameter-sensitivity tests, and cross-size evaluations further validate structured decoding and establish its operating scope.

Future work will improve the translation from planning allocations to executable radio resources and combine digital twins with short-horizon prediction for proactive response to dynamic demand. Security states and robust constraints will also be incorporated to address malicious interference and untrusted relays. For aerial–terrestrial 6G, the regional graph can be extended to a channel-model-driven time-varying 3-D representation with joint trajectory, association, and beam control.

## Figures and Tables

**Table 1:** Comparison of the principal methods on 100 shared test scenarios. Cells report means and 95% crossed training-seed–scenario bootstrap CIs. Bold indicates the highest mean for each metric.

**Panel A: Recovery Capability**			
**Method**	**Category**	**FinalCap**	**CTI**
**RLS-CAD**	Proposed	**0.644 [0.601, 0.685]**	**0.218 [0.197, 0.239]**
Graph-PPO	Dense graph policy	0.509 [0.471, 0.545]	0.083 [0.069, 0.098]
One-step Marginal Greedy	Heuristic	0.596 [0.551, 0.637]	0.170 [0.151, 0.189]
**Panel B: Utility and return**			
Method		TaskUtility	EpisodeReturn
**RLS-CAD**		**0.797 [0.761, 0.831]**	**0.165 [0.146, 0.185]**
Graph-PPO		0.704 [0.668, 0.738]	0.087 [0.070, 0.106]
One-step Marginal Greedy		0.743 [0.702, 0.780]	0.015 [−0.000, 0.030]
**Panel C: Allocation behavior**			
Method		EffectiveActiveRatio	SwitchCost
**RLS-CAD**		0.257 [0.190, 0.356]	0.215 [0.179, 0.255]
Graph-PPO		1.000 [1.000, 1.000]	0.029 [0.028, 0.031]
One-step Marginal Greedy		0.025 [0.024, 0.027]	1.415 [1.310, 1.515]

**Table 2:** Independent training-seed results for each learning method. Each cell reports FinalCap/TaskUtility over 100 shared test scenarios.

Method	Seed 0	Seed 1	Seed 2	Seed 3	Seed 4
RLS-CAD	0.6385/0.7898	0.6493/0.8019	0.6336/0.7931	0.6503/0.7987	0.6485/0.8022
Graph-PPO	0.4876/0.6941	0.5108/0.7204	0.5060/0.6890	0.5172/0.6983	0.5259/0.7186
RLS-CAD (Softmax Gate)	0.6274/0.7792	0.6417/0.7930	0.6453/0.7960	0.6474/0.7990	0.6439/0.7994
RLS-CAD (Softmax Allocation)	0.6123/0.7805	0.5976/0.7677	0.6075/0.7792	0.6037/0.7735	0.5878/0.7675
Signal-Matched Softmax	0.5148/0.6834	0.5827/0.7625	0.5903/0.7688	0.5498/0.7436	0.6147/0.7795
Signal-Matched Sparse Projection	0.6245/0.7881	0.6277/0.7859	0.6088/0.7774	0.6253/0.7873	0.6267/0.7890
RLS-CAD w/o Gate	0.6337/0.7886	0.6293/0.7888	0.6327/0.7909	0.6355/0.7908	0.6345/0.7900
RLS-CAD w/o Chain	0.6106/0.7814	0.6241/0.7909	0.6213/0.7892	0.5732/0.7552	0.6069/0.7840

**Table 3:** Paired differences between RLS-CAD and the controlled methods, with crossed-bootstrap 95% CIs and Holm-adjusted Wilcoxon tests. Positive differences favor RLS-CAD.

**Panel A: Effect Differences**			
**Control**	Δ **FinalCap [95% CI]**	Δ **TaskUtility [95% CI]**	Δ **EpisodeReturn [95% CI]**
Graph-PPO	0.1345 [0.1170, 0.1536]	0.0931 [0.0785, 0.1080]	0.0783 [0.0632, 0.0932]
RLS-CAD (Softmax Gate)	0.0030 [−0.0061, 0.0123]	0.0039 [−0.0038, 0.0130]	−0.0022 [−0.0122, 0.0094]
Signal-Matched Softmax	0.0734 [0.0438, 0.1071]	0.0498 [0.0243, 0.0840]	0.0370 [0.0113, 0.0714]
Signal-Matched Sparse Projection	0.0215 [0.0121, 0.0315]	0.0116 [0.0050, 0.0181]	0.0038 [−0.0046, 0.0115]
One-step Marginal Greedy	0.0477 [0.0390, 0.0567]	0.0545 [0.0461, 0.0628]	0.1504 [0.1413, 0.1593]
**Panel B: Significance tests**			
**Control**		**FinalCap Holm** p	**TaskUtility Holm** p
Graph-PPO		$7.89\times 10^{-30}$	$7.89\times 10^{-30}$
RLS-CAD (Softmax Gate)		0.023	0.011
Signal-Matched Softmax		$7.89\times 10^{-30}$	$7.89\times 10^{-30}$
Signal-Matched Sparse Projection		$2.61\times 10^{-17}$	$2.13\times 10^{-12}$
One-step Marginal Greedy		$3.79\times 10^{-21}$	$1.41\times 10^{-21}$

**Table 4:** Results for all 14 methods across five balanced disturbance profiles. Cells report means and 95% crossed training-seed–scenario bootstrap CIs; bold indicates the highest mean in each column.

**Panel A: FinalCap**					
**Method**	**Critical**	**Backhaul**	**Backlog**	**Distributed**	**Compound**
**RLS-CAD**	**0.794 [0.791, 0.796]**	0.763 [0.741, 0.781]	0.697 [0.693, 0.700]	0.744 [0.734, 0.753]	0.223 [0.208, 0.237]
Graph-PPO	0.621 [0.573, 0.662]	0.594 [0.575, 0.611]	0.632 [0.612, 0.651]	0.539 [0.512, 0.564]	0.162 [0.153, 0.173]
RLS-CAD (Softmax Gate)	0.786 [0.769, 0.796]	0.770 [0.752, 0.784]	0.682 [0.670, 0.693]	**0.759 [0.745, 0.769]**	0.209 [0.189, 0.230]
RLS-CAD (Softmax Allocation)	0.743 [0.731, 0.754]	0.726 [0.711, 0.740]	0.649 [0.620, 0.674]	0.706 [0.692, 0.718]	0.186 [0.169, 0.205]
Signal-Matched Softmax	0.740 [0.707, 0.766]	0.661 [0.610, 0.706]	0.661 [0.642, 0.679]	0.610 [0.534, 0.679]	0.180 [0.167, 0.193]
Signal-Matched Sparse Projection	0.778 [0.768, 0.789]	0.734 [0.720, 0.749]	0.674 [0.648, 0.694]	0.699 [0.681, 0.722]	0.228 [0.205, 0.253]
RLS-CAD w/o Gate	0.793 [0.792, 0.794]	**0.778 [0.772, 0.782]**	0.679 [0.666, 0.690]	0.739 [0.734, 0.743]	0.177 [0.167, 0.188]
RLS-CAD w/o Chain	0.751 [0.740, 0.759]	0.741 [0.720, 0.757]	0.618 [0.587, 0.646]	0.693 [0.635, 0.726]	**0.233 [0.217, 0.247]**
One-step Marginal Greedy	0.752 [0.748, 0.755]	0.713 [0.697, 0.727]	0.699 [0.696, 0.702]	0.656 [0.646, 0.665]	0.162 [0.148, 0.177]
Service-Deficit Greedy	0.738 [0.736, 0.741]	0.704 [0.696, 0.713]	0.562 [0.550, 0.573]	0.555 [0.536, 0.569]	0.143 [0.134, 0.152]
Greedy Bottleneck Relief	0.707 [0.699, 0.714]	0.704 [0.688, 0.718]	**0.699 [0.696, 0.702]**	0.636 [0.624, 0.648]	0.195 [0.185, 0.205]
Priority-TopK	0.520 [0.496, 0.547]	0.544 [0.527, 0.561]	0.562 [0.549, 0.575]	0.358 [0.337, 0.380]	0.153 [0.145, 0.161]
Priority-Proportional	0.532 [0.508, 0.560]	0.574 [0.556, 0.592]	0.608 [0.592, 0.624]	0.470 [0.443, 0.499]	0.179 [0.172, 0.186]
Uniform	0.512 [0.490, 0.536]	0.546 [0.528, 0.564]	0.601 [0.585, 0.617]	0.414 [0.388, 0.443]	0.151 [0.144, 0.159]
**Panel B: EpisodeReturn**					
**Method**	**Critical**	**Backhaul**	**Backlog**	**Distributed**	**Compound**
**RLS-CAD**	0.317 [0.307, 0.327]	0.186 [0.170, 0.202]	0.044 [0.037, 0.052]	0.176 [0.162, 0.190]	0.102 [0.086, 0.118]
Graph-PPO	0.224 [0.200, 0.250]	0.079 [0.062, 0.097]	0.028 [0.024, 0.032]	0.062 [0.045, 0.079]	0.042 [0.025, 0.060]
RLS-CAD (Softmax Gate)	0.316 [0.297, 0.331]	0.195 [0.181, 0.209]	**0.046 [0.039, 0.053]**	**0.199 [0.184, 0.212]**	0.081 [0.058, 0.104]
RLS-CAD (Softmax Allocation)	0.300 [0.290, 0.309]	0.166 [0.150, 0.181]	0.031 [0.023, 0.039]	0.156 [0.142, 0.170]	0.070 [0.053, 0.087]
Signal-Matched Softmax	0.291 [0.231, 0.326]	0.131 [0.088, 0.168]	0.040 [0.032, 0.048]	0.105 [0.051, 0.155]	0.074 [0.054, 0.093]
Signal-Matched Sparse Projection	0.323 [0.314, 0.331]	0.175 [0.161, 0.189]	0.040 [0.034, 0.048]	0.155 [0.135, 0.178]	0.113 [0.096, 0.133]
RLS-CAD w/o Gate	**0.324 [0.315, 0.332]**	**0.198 [0.186, 0.210]**	0.039 [0.028, 0.050]	0.188 [0.175, 0.200]	0.064 [0.050, 0.077]
RLS-CAD w/o Chain	0.309 [0.295, 0.321]	0.179 [0.159, 0.196]	0.022 [0.011, 0.033]	0.162 [0.120, 0.190]	**0.114 [0.099, 0.130]**
One-step Marginal Greedy	0.145 [0.133, 0.156]	0.035 [0.019, 0.052]	−0.073 [−0.080, −0.065]	−0.020 [−0.034, −0.008]	−0.014 [−0.022, −0.006]
Service-Deficit Greedy	0.300 [0.291, 0.308]	0.145 [0.133, 0.157]	0.000 [−0.001, 0.001]	0.051 [0.040, 0.062]	−0.007 [−0.012, −0.001]
Greedy Bottleneck Relief	0.046 [0.033, 0.058]	0.017 [0.003, 0.032]	−0.067 [−0.073, −0.060]	−0.037 [−0.052, −0.023]	−0.021 [−0.026, −0.015]
Priority-TopK	0.155 [0.140, 0.171]	0.041 [0.030, 0.052]	−0.000 [−0.001, −0.000]	−0.067 [−0.077, −0.059]	0.027 [0.021, 0.033]
Priority-Proportional	0.151 [0.138, 0.164]	0.073 [0.063, 0.084]	0.018 [0.017, 0.020]	0.022 [0.009, 0.034]	0.040 [0.034, 0.046]
Uniform	0.062 [0.057, 0.067]	0.033 [0.030, 0.036]	0.016 [0.014, 0.017]	−0.021 [−0.032, −0.010]	−0.013 [−0.016, −0.010]

**Table 5:** Exclusive batch-1 planning-inference benchmark. Values are post-warm-up median, p95, and p99 repeated timings; non-native projection stages are marked –. Actor device identifies model placement; the analytical decoder and NumPy projector remain on the host CPU. Repeated calls are not inferential samples, and the benchmark supports planning-cycle latency only, not 5G/6G physical-frame deadlines.

**Panel A: N = 180 Method and Stage Latency**											
**Method**	**Actor Device**	**Parameters**	**Action Dim.**	**Actor p50**	**Actor p95**	**Decoder p50**	**Decoder p95**	**Projector p50**	**Projector p95**	**Actor+Decoder p50**	**p95/p99 (ms)**
RLS-CAD	CPU	582,047	15	2.121	2.183	0.859	0.886	0.031	0.032	3.051	3.169/3.441
RLS-CAD	GPU	582,047	15	1.169	1.208	0.879	0.903	0.043	0.044	2.088	2.141/2.245
Graph-PPO	CPU	580,150	180	3.571	3.672	0.025	0.026	–	–	3.618	3.702/3.815
Graph-PPO	GPU	580,150	180	1.263	1.420	0.038	0.039	–	–	1.312	1.382/1.516
RLS-CAD (Softmax Allocation)	CPU	582,047	15	2.105	2.136	0.842	0.867	–	–	3.042	3.151/3.259
RLS-CAD (Softmax Allocation)	GPU	582,047	15	1.209	1.238	0.871	0.890	–	–	2.115	2.160/2.310
RLS-CAD (Softmax Gate)	CPU	582,047	15	2.111	2.267	0.846	0.877	0.031	0.032	3.032	3.172/3.551
RLS-CAD (Softmax Gate)	GPU	582,047	15	1.208	1.233	0.875	0.899	0.044	0.045	2.122	2.167/2.316
Signal-Matched Softmax	CPU	580,918	180	3.598	3.694	0.054	0.058	–	–	3.690	3.770/3.894
Signal-Matched Softmax	GPU	580,918	180	1.239	1.275	0.069	0.072	–	–	1.330	1.363/1.469
Signal-Matched Sparse Projection	CPU	580,918	180	3.577	3.648	0.071	0.072	0.031	0.031	3.716	3.793/3.902
Signal-Matched Sparse Projection	GPU	580,918	180	1.256	1.290	0.088	0.090	0.043	0.044	1.364	1.392/1.503
**Panel B: RLS-CAD Cross-Size Latency and Memory**											
**Actor Device**			**N**		**p50**	**p95**	**p99**	**Environment MiB**		**Peak GPU MiB**	
CPU			80		1.998	2.050	2.187	0.11		0.0	
CPU			180		3.072	3.190	3.327	0.53		0.0	
CPU			270		4.477	4.634	4.886	1.16		0.0	
GPU			80		2.019	2.088	2.222	0.11		18.6	
GPU			180		2.147	2.206	2.386	0.53		16.5	
GPU			270		2.293	2.398	2.709	1.16		19.8	

## Data Availability

Code, data, and analysis materials for this study are available in GitHub Release v1.0.0 (https://github.com/MurrayMa0816/rls_cad/releases/tag/v1.0.0, accessed on 17 August 2026).

## Appendix A. Self-Contained Reproducibility Specification

This appendix provides the implementation details referenced by the main text. Appendices Appendix A.1, Appendix A.2 and Appendix A.3 define the simulator, Appendix A.4 specifies the policy and network implementation, and Appendix A.5 records the reference configuration, software environment, and statistical settings. All clipping is componentwise.

### Appendix A.1. Computational Roles and Typed Graph

#### Appendix A.1.1. Role Assignment and Display Mapping

Index a region by row–column coordinates $\left(r_{i},c_{i}\right)$ with zero-based indices and center $\left(r_{0},c_{0}\right)=\left(\left(R-1\right)/2,\left(C-1\right)/2\right)$. The nine computational roles, in fixed order, are airfield, access, transfer, fuel, power, comm, repair, assembly, and buffer. Role assignment is deterministic and sequential: (i) initialize all regions as buffer; (ii) assign airfield when $|r_{i}-r_{0}|\leq \max\left(1,0.08R\right)$ and $|c_{i}-c_{0}|\leq \max\left(1,0.10C\right)$; (iii) assign non-core regions as access when $|r_{i}-r_{0}|\leq 1$ or $|c_{i}-c_{0}|\leq 1$; (iv) overwrite as transfer when $(c_{i}<0.18C$ or $c_{i}>0.82C)$ and $|r_{i}-r_{0}|\leq 1$; and (v) assign non-route regions as assembly when $\left(r_{i}>0.72R,c_{i}>0.65C\right)$ or $\left(r_{i}<0.25R,c_{i}<0.22C\right)$. From the remaining non-core, non-route regions within 0.55 of the maximum center distance, let C be the row-major candidate list and s=max(1,⌊|C|/5⌋). For j=0,1,2,3, the first max(2,⌊N/80⌋) entries of C[js::3s] are assigned, respectively, to fuel, power, comm, and repair. The default role counts are (6,44,10,1,2,2,1,27,87).

The six labels in Figure 1 are display aggregates: airfield↦network entry; access/repair↦temporary access; transfer/fuel/comm↦backhaul relay; power↦priority access; assembly↦remote demand; and buffer↦general affected area. They do not replace the nine computational roles. The displayed hexagons are visualization units; the computational spatial graph is the four-neighbor row–column lattice.

#### Appendix A.1.2. Typed-Edge Construction

An undirected spatial edge joins horizontal or vertical neighbors. An undirected route edge additionally requires both endpoints to have roles in {airfield,access,transfer,assembly}. A directed support edge $A_{ij}^{\sup}=1$ points from provider *i* to target *j*. Targets have roles in {airfield,assembly,transfer}; for each target and for each provider class in {fuel,power,comm,repair,access}, the three nearest providers of that class (or all if fewer than three exist) are connected using normalized-coordinate Euclidean distance. Equal distances follow the numpy.argsort order under the locked NumPy 2.1.2 environment; the release also provides the resulting role array and all three adjacency matrices. The default graph has 333 undirected spatial edges, 132 undirected route edges, and 387 directed support edges. All boundary regions

$$E=\{i:r_{i}\in \left\{0,R-1\right\}\;\mathrm{or}\;c_{i}\in \left\{0,C-1\right\}\}$$

are widest-path reachability anchors. This boundary set is distinct from the airfield computational role and the displayed network-entry label.

#### Appendix A.1.3. Static Regional Attributes

Let $d_{i}^{c}$ be center distance divided by its grid maximum and let $r_{i}$ be the role index. The role-ordered base-value and criticality vectors are

$$\bar{v}=\left(1,0.62,0.75,0.58,0.60,0.57,0.52,0.70,0.22\right),\;\bar{\chi }=\left(1,0.58,0.72,0.70,0.72,0.68,0.64,0.62,0.12\right).$$

With route degree $d_{i}^{\mathrm{route}}=\sum_{j}A_{ij}^{\mathrm{route}}$, regional value and criticality are

(A1) $$\begin{array}{cc} v_{i} & ={\bar{v}}_{r_{i}}\left(0.75+0.25\left(1-d_{i}^{c}\right)\right), \end{array}$$

(A2) $$\begin{array}{cc} \chi_{i} & ={\bar{\chi }}_{r_{i}}. \end{array}$$

Route incidence and repair efficiency are

(A3) $$\begin{array}{cc} b_{i}^{\mathrm{route}} & =d_{i}^{\mathrm{route}}/\max_{j}d_{j}^{\mathrm{route}}, \end{array}$$

(A4) $$\begin{array}{cc} e_{i}^{\mathrm{rep}} & =\mathrm{clip}(0.65+0.25I_{r_{i}=\mathrm{repair}}+0.10b_{i}^{\mathrm{route}},0.2,1). \end{array}$$

The dependency, queue, repair-scale, and placement-cost quantities are

(A5) $$\begin{array}{cc} \zeta_{i} & =\mathrm{clip}\;\left(\frac{\sum_{j}A_{ji}^{\sup}}{4},0,1\right), \end{array}$$

(A6) $$\begin{array}{cc} {\bar{Q}}_{i} & =\mathrm{clip}(0.5+0.7v_{i}+0.3\chi_{i},0.4,1.6), \end{array}$$

(A7) $$\begin{array}{cc} s_{i}^{\mathrm{rep}} & =\mathrm{clip}(0.12+0.30\left(1-e_{i}^{\mathrm{rep}}\right),0.08,0.5), \end{array}$$

(A8) $$\begin{array}{cc} c_{i}^{\mathrm{exec}} & =\mathrm{clip}(0.15+0.40d_{i}^{c}+0.25\left(1-b_{i}^{\mathrm{route}}\right),0.05,1). \end{array}$$

The quantity denoted $b_{i}^{\mathrm{corr}}$ in Equation (27) is a fixed corridor-centrality proxy. Specifically,

(A9) $$\begin{array}{cc} {\tilde{b}}_{i}^{\mathrm{corr}} & =\max\;\left\{e^{-|r_{i}-r_{0}|/\max\left(0.15R,1\right)},e^{-|c_{i}-c_{0}|/\max\left(0.15C,1\right)}\right\}\left[0.4+0.6I\left(d_{i}^{\mathrm{route}}>0\right)\right], \end{array}$$

(A10) $$\begin{array}{cc} b_{i}^{\mathrm{corr}} & ={\tilde{b}}_{i}^{\mathrm{corr}}/\max_{j}{\tilde{b}}_{j}^{\mathrm{corr}}. \end{array}$$

### Appendix A.2. Reset, Initial Distributions, and Profiles

#### Appendix A.2.1. Reset and Initial Conditions

Each episode seed is split with SeedSequence.spawn(3) into independent scenario, arrival, and transition-disturbance random streams. Reset sets t=0, $a_{-1}=0$, $g_{0}=Q_{0}=0$, $\eta_{0}^{\mathrm{loc}}=\kappa_{0}^{\sup}=1$, and every route-arc health $h_{0,ij}^{\mathrm{route}}=1$. It then samples regional readiness as

(A11) $$r_{0,i}^{\mathrm{ready}}∼U\left(0.75,1\right).$$

Regional demand is generated by

(A12) $$\begin{array}{cc} d_{0,i}^{\mathrm{demand}} & =\mathrm{clip}(0.45+0.55v_{i}+0.15Z_{i},0,1), \end{array}$$

(A13) $$\begin{array}{cc} Z_{i} & ∼N(0,1), \end{array}$$

All draws in Equations (A11)–(A13) are independent across regions. Before a new profile is applied, reset sets $\eta_{0}^{\mathrm{loc}}$ and $\kappa_{0}^{\sup}$ to one. The simulator then recomputes local support and route capability and evaluates $C_{0}$ and the four losses. This ordering prevents either array from carrying state across episodes and is used for all reported results.

#### Appendix A.2.2. Disturbance Profiles

The loss-weight order is (critical,support,backlog,e2e). Profile sampling is without replacement within each listed candidate set. Table A1 gives the complete profile-selection and state-modification rules.

**Table A1:** Default profile rules for N=180. Here U(a,b) denotes an independent uniform draw and “route arcs” counts directed nonzero entries of $A^{\mathrm{route}}$.

Profile and Loss Weights	Selected Regions	State Modification
Key damage (0.48,0.12,0.08,0.32)	10 regions with $\chi_{i}\geq 0.70$	$g_{i}∼U\left(0.65,1\right)$
Route fracture (0.08,0.50,0.08,0.34)	12 regions with route degree >0; 71 of 264 route arcs	$g_{i}∼U\left(0.45,0.85\right)$; $h_{ij}^{\mathrm{route}}∼U\left(0.10,0.50\right)$
Backlog (0.08,0.08,0.50,0.34)	14 repair/airfield/assembly regions	$Q_{i}={\bar{Q}}_{i}U\left(0.65,1\right)$; $r_{i}^{\mathrm{ready}}∼U\left(0.02,0.35\right)$
Distributed (0.14,0.14,0.20,0.52)	54 of all regions	$g_{i}∼U\left(0.20,0.50\right)$
Compound (0.14,0.18,0.12,0.56)	all four preceding region selections; 58 route arcs; 12 support providers	preceding updates, then $g_{i}\leftarrow \max\left(g_{i},U\left(0.40,0.78\right)\right)$, $Q_{i}\leftarrow \max\left(Q_{i},{\bar{Q}}_{i}U\left(0.35,0.78\right)\right)$, $r_{i}^{\mathrm{ready}}\leftarrow \min\left(r_{i}^{\mathrm{ready}},U\left(0.05,0.45\right)\right)$ for the 12 providers

During training, the probabilities for key, route, backlog, distributed, and compound profiles are (0.20,0.22,0.20,0.16,0.22). Formal evaluation uses mixed_balanced: the profile is selected by evaluation seed modulo five in the same order, so seeds 20001–20100 contain exactly 20 scenarios of each profile.

### Appendix A.3. Complete State Transition

#### Appendix A.3.1. Local State Dynamics

The default budget-dilution threshold is zero, so the effective intervention is $e_{t,i}=a_{t,i}$. The repair response is

(A14) $$\phi_{t,i}=\min\;\left(\frac{e_{t,i}}{s_{i}^{\mathrm{rep}}+\varepsilon },1\right)e_{i}^{\mathrm{rep}}\left(0.5+0.5r_{t,i}^{\mathrm{ready}}\right),$$

The four state-specific gains are

(A15) $$\begin{array}{cc} G_{i}^{g} & =0.35+0.45K_{i}+0.20\chi_{i}, \end{array}$$

(A16) $$\begin{array}{cc} G_{i}^{Q} & =0.24+0.52S_{i}+0.24I_{r_{i}=\mathrm{repair}}, \end{array}$$

(A17) $$\begin{array}{cc} G_{i}^{r} & =0.20+0.45S_{i}+0.25P_{i}+0.10\zeta_{i}, \end{array}$$

(A18) $$\begin{array}{cc} G_{i}^{h} & =0.18+0.58R_{i}+0.24b_{i}^{\mathrm{corr}}. \end{array}$$

where $K_{i}=I_{r_{i}\in \left\{\mathrm{airfield},\mathrm{assembly},\mathrm{transfer}\right\}}$, $S_{i}=I_{r_{i}\in \left\{\mathrm{fuel},\mathrm{power},\mathrm{comm},\mathrm{repair}\right\}}$, $P_{i}=I\left(\sum_{j}A_{ij}^{\sup}>0\right)$, and $R_{i}=I_{r_{i}\in \left\{\mathrm{airfield},\mathrm{access},\mathrm{transfer}\right\}}\left(0.45+0.55b_{i}^{\mathrm{route}}\right)$.

The state update is executed in the following order: Interruption, traffic arrival, and backlog are updated sequentially as

(A19) $$\begin{array}{cc} g_{t+1,i} & =\mathrm{clip}(g_{t,i}+\xi_{t,i}^{g}-0.44G_{i}^{g}\phi_{t,i},0,1), \end{array}$$

(A20) $$\begin{array}{cc} A_{t,i}^{\mathrm{arr}} & =0.06g_{t+1,i}M_{t,i}, \end{array}$$

(A21) $$\begin{array}{cc} Q_{t+1,i} & =\mathrm{clip}(Q_{t,i}+A_{t,i}^{\mathrm{arr}}+\xi_{t,i}^{Q}-0.32G_{i}^{Q}\phi_{t,i}r_{t,i}^{\mathrm{ready}},0,{\bar{Q}}_{i}). \end{array}$$

Readiness and route-arc health are then updated as

(A22) $$\begin{array}{cc} r_{t+1,i}^{\mathrm{ready}} & =\mathrm{clip}\;\left(r_{t,i}^{\mathrm{ready}}+0.16G_{i}^{r}e_{t,i}+0.08G_{i}^{r}\phi_{t,i}-0.04\frac{Q_{t+1,i}}{{\bar{Q}}_{i}+\varepsilon },0,1\right), \end{array}$$

(A23) $$\begin{array}{cc} h_{t+1,ij}^{\mathrm{route}} & =\mathrm{clip}\;\left(h_{t,ij}^{\mathrm{route}}+0.10\left(G_{i}^{h}e_{t,i}+G_{j}^{h}e_{t,j}\right),0,1\right). \end{array}$$

In the main experiment, $\xi_{t,i}^{g}=\xi_{t,i}^{Q}=0$ and $M_{t,i}=1$. When the optional stochastic-disturbance flag is enabled, $\xi_{t,i}^{g},\xi_{t,i}^{Q}\overset{\mathrm{iid}}{∼}N\left(0,0.01^{2}\right)$. For an arrival coefficient of variation c≥0, the sensitivity experiment uses

(A24) $$M_{t,i}=\exp\left(sZ_{t,i}-s^{2}/2\right),\;s=\sqrt{\log(1+c^{2})},\;Z_{t,i}\overset{\mathrm{iid}}{∼}N\left(0,1\right),$$

which has unit mean and reduces to one at the default value c=0.

#### Appendix A.3.2. Support and Route-Capability Updates

Let $o_{t+1,i}=1-g_{t+1,i}$ and retain the preceding local-support value in the provider-service term: Provider service and its incoming aggregation are

(A25) $$\begin{array}{cc} y_{t+1,j} & =o_{t+1,j}\eta_{t,j}^{\mathrm{loc}}r_{t+1,j}^{\mathrm{ready}}\left(1-\frac{Q_{t+1,j}}{{\bar{Q}}_{j}+\varepsilon }\right), \end{array}$$

(A26) $$\begin{array}{cc} {\bar{y}}_{t+1,i} & =\left\{\begin{array}{cc} \frac{\sum_{j}A_{ji}^{\sup}y_{t+1,j}}{\sum_{j}A_{ji}^{\sup}+\varepsilon }, & \sum_{j}A_{ji}^{\sup}>0,\\ 1, & \mathrm{otherwise}, \end{array}\right. \end{array}$$

The aggregation determines local support, route-edge weight, and widest-path capability:

(A27) $$\begin{array}{cc} \eta_{t+1,i}^{\mathrm{loc}} & =o_{t+1,i}\left[\left(1-\zeta_{i}\right)+\zeta_{i}{\bar{y}}_{t+1,i}\right], \end{array}$$

(A28) $$\begin{array}{cc} W_{t+1,ij} & =A_{ij}^{\mathrm{route}}h_{t+1,ij}^{\mathrm{route}}\min\left\{o_{t+1,i}\eta_{t+1,i}^{\mathrm{loc}},o_{t+1,j}\eta_{t+1,j}^{\mathrm{loc}}\right\}, \end{array}$$

(A29) $$\begin{array}{cc} \kappa_{t+1,i}^{\sup} & =\max_{p\in P(E,i)}\;\min_{(j,k)\in p}W_{t+1,jk}. \end{array}$$

The widest-path value is zero when no path exists and one for a length-zero path at a boundary anchor. The reset-sampled demand is fixed within an episode, $d_{t+1,i}^{\mathrm{demand}}=d_{t,i}^{\mathrm{demand}}$, as are the graph, roles, and static regional attributes. Finally, the simulator increments time, recomputes Equation (6), the four losses in Equation (7), and the reward in Equation (9), and stores $a_{t}$ and $\left\{L_{t}^{k}\right\}$ as the previous-allocation and previous-loss variables for the next decision. Together with these carry-forward rules, reset distributions, and profile table, Equations (A14)–(A29) define the transition *T* in Equation (5).

### Appendix A.4. Policy and Network Implementation

#### Appendix A.4.1. Policy Inputs and Graph Preprocessing

Let ${\mathrm{onehot}}_{9}\left(r_{i}\right)$ denote the nine-component role indicator. The regional and global policy inputs are, in their implemented order,

(A30) $$\begin{array}{cc} x_{t,i}^{\mathrm{node}}= & [{\mathrm{onehot}}_{9}\left(r_{i}\right),r_{i}/\left(R-1\right),c_{i}/\left(C-1\right),v_{i},\chi_{i},e_{i}^{\mathrm{rep}},\zeta_{i},{\bar{Q}}_{i},s_{i}^{\mathrm{rep}},\\ \; & g_{t,i},\kappa_{t,i}^{\sup},\eta_{t,i}^{\mathrm{loc}},r_{t,i}^{Q},r_{t,i}^{\mathrm{ready}},d_{t,i}^{\mathrm{demand}},a_{t-1,i}/B]\in R^{24}. \end{array}$$

(A31) $$x_{t}^{\mathrm{global}}=\left[t/H,C_{t},L_{t}^{\mathrm{critical}},L_{t}^{\mathrm{support}},L_{t}^{\mathrm{backlog}},L_{t}^{e2e},\omega_{1},\omega_{2},\omega_{3},\omega_{4}\right]\in R^{10}.$$

where $r_{t,i}^{Q}=\mathrm{clip}\left(Q_{t,i}/\left({\bar{Q}}_{i}+\varepsilon \right),0,1\right)$. Static quantities in Equations (A30) and (A31) are defined in Equations (A1)–(A8); all remaining entries are current state variables or the preceding allocation.

The encoder first symmetrizes only the directed support relation:

(A32) $$\begin{array}{cc} {\hat{A}}^{\mathrm{sp}} & =A^{\mathrm{sp}}, \end{array}$$

(A33) $$\begin{array}{cc} {\hat{A}}^{\mathrm{route}} & =A^{\mathrm{route}}, \end{array}$$

(A34) $$\begin{array}{cc} {\hat{A}}^{\sup} & =\max(A^{\sup},{\left(A^{\sup}\right)}^{⊤}). \end{array}$$

It then row-normalizes each relation:

(A35) $$A_{ij}^{r,\mathrm{enc}}=\frac{{\hat{A}}_{ij}^{r}}{\max(\sum_{h}{\hat{A}}_{ih}^{r},1)}.$$

Thus, support-edge symmetrization precedes normalization and a zero-degree row remains zero. For relation r∈{sp,route,sup}, each typed message-passing layer first aggregates

(A36) $$m_{t,i}^{\left(l,r\right)}=\sum_{j}A_{ij}^{r,\mathrm{enc}}W_{r}^{\left(l\right)}h_{t,j}^{\left(l\right)}.$$

The layer then updates each regional representation as

(A37) $$h_{t,i}^{\left(l+1\right)}=\mathrm{ReLU}\;\left(W_{s}^{\left(l\right)}h_{t,i}^{\left(l\right)}+\sum_{r}m_{t,i}^{\left(l,r\right)}\right).$$

#### Appendix A.4.2. Reference Decoder Configuration

The main text defines the decoder structure independently of a particular numerical setting. Table A2 groups the fixed reference configuration by function. The first block specifies signal aggregation and actor-conditioned gating; the second block contains benchmark scaling and cost conventions. These settings instantiate the reported benchmark rather than alter the decoder topology, and none varies across training seeds or test scenarios.

**Table A2:** Reference decoder configuration used in the reported experiments. Symbols follow Equations (11)–(33).

Configuration Group	Symbol	Fixed Setting
Decoder-defining settings		
Pressure aggregation	$\beta_{m}$	(0.55,0.30,0.15)
Gate scaling	$\omega_{\min},\omega_{\mathrm{ref}},\left(\lambda_{u},\lambda_{m},\lambda_{\omega }\right)$	0.03,0.25,(0.25,0.20,0.18)
Gate coupling	*M*	$\left[\begin{array}{cccc} 1 & 0.08 & 0.04 & 0.18\\ 0.08 & 1 & 0.06 & 0.18\\ 0.05 & 0.05 & 0.45 & 0.20\\ 0.18 & 0.22 & 0.55 & 1 \end{array}\right]$
Local recovery gap	$\beta_{\mathrm{gap}}$	(0.45,0.25,0.20,0.10)
Primary score maps	$\beta_{\mathrm{critical}},\beta_{\sup},\beta_{\mathrm{back}}$	(0.50,0.50),(1,0.60,0.35),(0.80,0.45,0.25)
End-to-end components	$\beta_{\mathrm{bridge}},\beta_{\mathrm{anchor}},\beta_{e2e}$	(0.35,0.65),(0.40,0.32,0.28),(0.46,0.20,0.22,0.12)
Benchmark scaling and cost settings		
Pressure references	$L_{\min},m_{\max},s$	0.03,3,(0.28,0.18,0.10,0.34)
Placement cost	$\beta_{c},c_{\min},c_{\max}$	(0.15,0.40,0.25),0.05,1

#### Appendix A.4.3. Actor-Control Transformations

The sampled policy control $u_{t}$ in Equation (15) is the stochastic variable used in the PPO likelihood and entropy calculations. Before deterministic decoding, the environment clips each coordinate to [−8,8] and applies the transformations in Table A3. The channel index in the last two rows is k∈{1,2,3,4}.

**Table A3:** Coordinate-wise mapping from the clipped policy control to the decoder variables. Approximate induced ranges include the input bound [−8,8]; the transformations are exact.

Coordinates	Decoder Variable	Transformation and Induced Range
1:4	gate logits	$0.25{\tilde{u}}_{t,1:4}\in {\left[-2,2\right]}^{4}$ enters Equation (18); $\alpha_{t}\in \Delta^{3}$
5	projection temperature	$\tau_{t}=1.25\left[0.06+0.44\mathrm{sigmoid}\left({\tilde{u}}_{t,5}\right)\right]\in \left[0.075,0.625\right]$
6	score scale	$\gamma_{t}=\mathrm{softplus}\left({\tilde{u}}_{t,6}-0.75\right)\in \left[1.58\;\times \;10^{-4},7.251\right]$
7	execution-cost scale	$\mu_{t}=0.20\mathrm{softplus}\left({\tilde{u}}_{t,7}-2.50\right)\in \left[5.51\;\times \;10^{-6},1.101\right]$
8:11	channel gains	$\nu_{t,k}=1+1.30\left[\mathrm{sigmoid}\left({\tilde{u}}_{t,7+k}\right)-0.5\right]\in \left[0.350,1.650\right]$
12:15	residual coefficients	$\varrho_{t,k}=0.25\tanh\left({\tilde{u}}_{t,11+k}\right)\in \left[-0.25,0.25\right]$

#### Appendix A.4.4. Network Architecture

Table A4 lists the complete policy-network architecture. The actor and critic branches do not share their post-fusion multilayer perceptrons. The policy log standard deviation is a learned, state-independent 15-component vector. The complete model has 582,047 trainable parameters.

**Table A4:** Policy-network architecture used in all reported RLS-CAD runs.

Component	Dimensions	Activation or Operation
Regional preprocessing	24→128→128	ReLU after each layer
Typed graph encoder	two 128→128 layers	Equations (A36) and (A37); ReLU
Graph pooling	128+128	regional mean and maximum
Global branch	10→128	ReLU
Fusion	384→256	concatenation followed by ReLU
Actor branch	256→256→256→128	Tanh after each hidden layer
Actor outputs	mean 128→15; log standard deviation 15	linear mean head; learned parameter vector
Critic branch	256→256→256→128→1	Tanh hidden layers; linear value head

### Appendix A.5. Implementation and Statistical Settings

#### Appendix A.5.1. Reference Experiment Configuration

The reported experiments use R=12, C=15, N=RC=180, horizon H=40, and budget B=1. Shared numerical settings are $\lambda_{\zeta }=1.5$ and $\varepsilon =10^{-8}$; the reward and reporting conventions use $\eta_{\mathrm{sw}}=0.002$, $\eta_{\mathrm{act}}=0$, and $a_{\min}=10^{-3}B$. These values define the reference experiment rather than the RLS-CAD architecture.

#### Appendix A.5.2. Statistical Analysis and Software Environment

The main crossed training-seed–scenario bootstrap uses 20,000 replicates with random seed 20260731. Paired Wilcoxon tests operate on the 100 scenario means, and Holm adjustment is applied within each metric family. The switching-condition analysis uses 5000 bootstrap replicates with seed 20260801. Other auxiliary analyses use the replicate counts and seeds recorded in their released manifests.

Table A5 summarizes the environment recorded for all 40 reported learning runs. The release cited in the Data Availability Statement contains the exact dependency specification, seed and configuration records, raw main and auxiliary results, and analysis scripts.

**Table A5:** Recorded software and compute environment for the reported learning runs.

Item	Recorded Version
Python	3.11.10
NumPy	2.1.2
PyTorch	2.4.0+cu121
Gymnasium	1.2.3
Stable-Baselines3	2.8.0
PyYAML	6.0.2
CUDA/GPU	CUDA 12.1/NVIDIA GeForce RTX 3090
